# *Orientia tsutsugamushi* selectively stimulates the C-type lectin receptor Mincle and type 1-skewed proinflammatory immune responses

**DOI:** 10.1371/journal.ppat.1009782

**Published:** 2021-07-28

**Authors:** James Fisher, Galen Card, Yuejin Liang, Brandon Trent, Holly Rosenzweig, Lynn Soong

**Affiliations:** 1 Department of Microbiology and Immunology, University of Texas Medical Branch, Galveston, Texas, United States of America; 2 Department of Pathology, University of Texas Medical Branch, Galveston, Texas, United States of America; 3 Department of Medicine, Division of Rheumatology, University of Colorado Anschutz Medical Campus, Aurora, Colorado, United States of America; 4 School of Medicine, Program in Molecular and Cellular Biosciences, Oregon Health and Science University, Portland, Oregon, United States of America; Johns Hopkins University, UNITED STATES

## Abstract

*Orientia tsutsugamushi* is an obligately intracellular bacterium and the etiological agent of scrub typhus. The lung is a major target organ of infection, displaying type 1-skewed proinflammatory responses. Lung injury and acute respiratory distress syndrome are common complications of severe scrub typhus; yet, their underlying mechanisms remain unclear. In this study, we investigated whether the C-type lectin receptor (CLR) Mincle contributes to immune recognition and dysregulation. Following lethal infection in mice, we performed pulmonary differential expression analysis with NanoString. Of 671 genes examined, we found 312 significantly expressed genes at the terminal phase of disease. Mincle (*Clec4e*) was among the top 5 greatest up-regulated genes, accompanied with its signaling partners, type 1-skewing chemokines (*Cxcr3*, *Ccr5*, and their ligands), as well as *Il27*. To validate the role of Mincle in scrub typhus, we exposed murine bone marrow-derived macrophages (MΦ) to live or inactivated *O*. *tsutsugamushi* and analyzed a panel of CLRs and proinflammatory markers via qRT-PCR. We found that while heat-killed bacteria stimulated transitory Mincle expression, live bacteria generated a robust response in MΦ, which was validated by indirect immunofluorescence and western blot. Notably, infection had limited impact on other tested CLRs or TLRs. Sustained proinflammatory gene expression in MΦ (*Cxcl9*, *Ccl2*, *Ccl5*, *Nos2*, *Il27*) was induced by live, but not inactivated, bacteria; infected Mincle^-/-^ MΦ significantly reduced proinflammatory responses compared with WT cells. Together, this study provides the first evidence for a selective expression of Mincle in sensing *O*. *tsutsugamushi* and suggests a potential role of Mincle- and IL-27-related pathways in host responses to severe infection. Additionally, it provides novel insight into innate immune recognition of this poorly studied bacterium.

## Author summary

Scrub typhus is a life-threatening disease caused by the bacterium *Orientia tsutsugamushi*. Severe disease often involves the lung, where diffuse alveolar damage can lead to significant morbidity and mortality. It is thought that dysregulated immune responses contribute to the development of severe disease; however, it remains unknown as to how the immune system senses and responds to *O*. *tsutsugamushi*. In this study, we used a lethal mouse model of scrub typhus, along with cultured macrophages and neutrophils, to examine immune sensors and inflammatory responses which may be involved during infection. We found that *O*. *tsutsugamushi* selectively stimulated the C-type lectin receptor Mincle in the lungs of infected mice, which was accompanied by increased markers of proinflammatory and type 1-responses. We also found that macrophages treated with live or inactivated bacteria increased Mincle expression alongside type 1 and M1 proinflammatory markers, some of which were markedly decreased in Mincle^-/-^ cells. Activation of Mincle and its associated inflammatory profile by both live and inactivated *O*. *tsutsugamushi* may generate an overzealous immune response that contributes to acute tissue damage. This study provides novel insight into immune recognition of *O*. *tsutsugamushi* and a new framework for evaluating immune dysregulation in scrub typhus.

## Introduction

Scrub typhus is a vector-borne febrile illness caused by the obligately intracellular bacterium, *Orientia tsutsugamushi*. Transmitted via the bite of a larval *Leptotrombidium* mite (commonly known as chigger), this bacterium infects approximately 1 million people per year in an Asia-Pacific region housing over one-third of the world’s population, termed the “tsutsugamushi triangle” [1]. Recent reports have indicated the presence of scrub typhus in areas previously thought free of the disease, including South America and Africa [2,3]. The lung is a major target organ of infection, and mild interstitial pneumonia predominates in self-limiting or appropriately treated cases [1]. However, if left untreated, disease may progress to severe lung damage and acute respiratory distress syndrome in up to 25% of cases [1,4]. While the facets underpinning progression from mild to severe disease remain ill-defined, both bacterial and host factors are speculated to play major roles.

The bacterial factors responsible for scrub typhus pathogenesis remain elusive. *O*. *tsutsugamushi* is remarkably unique in that over 40% of its genome consists of repeated DNA sequences, including transposable and massively amplified integrative and conjugated elements, as well as short repetitive sequences [5,6]. These elements have rendered attempts at genetic manipulation unsuccessful, precluding functional genomic studies and the generation of fluorescently tagged “trackable” bacteria [7]. Additionally, this bacterium lacks lipopolysaccharide and conventional peptidoglycan structures, which is a distinguishing feature from the closely related *Rickettsia* genus [8]. *O*. *tsutsugamushi* preferentially replicates within endothelial cells, monocytes, macrophages (MΦ), and dendritic cells [9,10]. After gaining entrance to host cells via the phagosome or endosome, the bacteria escape to the cytoplasm for replication. Following replication, bacteria disseminate by budding from the host cell cytoplasmic membrane [11]. Endothelial and MΦ responses to infection have been characterized and include detection of proinflammatory cytokines (IL-1β, TNF-α, IL-8) and activation of transcriptional factor NF-κB [12,13]. The MΦ response to *O*. *tsutsugamushi* has garnered research interest both in terms of being an innate immune responder and as a host cell to infection. *In-vitro* studies with human primary monocytes/MΦ have indicated the generation of antivirus-like immune responses shortly after exposure to *O*. *tsutsugamushi*, as well as a skewed proinflammatory (M1) phenotype in MΦ [14]. The biological roles of tissue-specific MΦ subsets in scrub typhus cases remain unexplored, although studies examining human tissue samples have been reported [10,15].

Animal models of scrub typhus, which mimic pathology observed in human patients, have shed light on possible mechanisms of pathogenesis and tissue-specific immune responses. Our recent studies with lethal *O*. *tsutsugamushi* infection in mice have revealed strong, type 1-skewed immune responses in the lung, spleen, liver, kidney, and brain tissues [16,17]. Since the lungs harbor the greatest bacterial burden throughout the course of infection, we recently examined the activation status of MΦ, neutrophils, and endothelial cells in the lungs of infected mice [18]. We observed a significant influx of monocytes/MΦs on day 6 post-infection (onset of disease) and day 10 post-infection (prior to host death), with nearly the entire MΦ population (~97% cells) skewed towards the M1 phenotype at the terminal phase of disease (day 10), implying that M1 MΦs confer ineffective protection and could play a role in tissue injury [18]. *In-vitro* experiments with murine bone marrow-derived MΦs revealed restricted *O*. *tsutsugamushi* growth in M1-polarized MΦs, but unrestricted growth in naïve and M2-polarized MΦs [18]. Collectively, these studies alluded to the double-edged sword of M1 MΦs in scrub typhus, suggesting that while M1 polarization contributes to controlling *O*. *tsutsugamushi* infection, the dysregulation of this response could lead to indiscriminate tissue damage. However, the initial driving factors for M1 MΦ polarization remain unknown.

Pathogen pattern recognition receptors (PRRs), including Toll-like receptors (TLRs), nucleotide-binding oligomerization domain-like receptors, retinoic acid-inducible gene-1 like receptors, and C-type lectin receptors (CLRs), are crucial for initiating and shaping immune responses to infection. CLRs orchestrate inflammatory responses via an immunoreceptor tyrosine-based inhibitory motif (ITIM) or immunoreceptor tyrosine-based activation motif (ITAM) in its own cytoplasmic tail, or through coupling with ITIM or ITAM-bearing signaling partners [19–21]. Mincle (macrophage inducible C-type lectin; *Clec4e* or *Clecsf9*) is expressed mostly on myeloid cells and has been studied extensively for its contribution to MΦ activation and skewing innate and adaptive responses. Mincle can bind pathogen-associated glycolipids, as well as host damage-associated molecular patterns (DAMPs) such as cholesterol crystals (in humans) and SAP130 (in humans and mice) [22–24]. Since Mincle does not harbor an ITIM or ITAM, it relies on Fc receptor gamma (*Fcgr;* FcγR) to initiate immune signaling cascades [24]. While the functional significance of Mincle activation is variable in different host-pathogen interactions or diseases, Mincle signaling is known to lead to proinflammatory cytokine production, M1 MΦ phenotype, and a type 1- or Th17-favoring cytokine milieu [23,25,26]. To date, CLRs have been virtually unstudied in the context of obligately intracellular pathogens, and the PRR profile responding to *O*. *tsutsugamushi* remains poorly characterized.

In this study, we tested whether CLRs, particularly Mincle, could contribute to the dysregulated type-1 response to *O*. *tsutsugamushi*. First, we utilized a lethal infection model in C57BL/6 mice to show that Mincle and its signaling partners, *Fcgr*, were highly differentially expressed in the lung tissues during scrub typhus, with the greatest mRNA and protein levels towards D10 (the terminal phase of disease prior to host death). The Mincle and *Fcgr* levels positively correlated with the expression levels of proinflammatory cytokines/chemokines and M1 polarization markers. Furthermore, we utilized bone marrow-derived MΦ from wild-type (WT) and Mincle-deficient (Mincle^-/-^) mice to demonstrate a selective activation of Mincle and an M1 transcriptional profile, but not other CLRs or type 2 markers, via interaction with live bacteria. To our knowledge, this is the first report defining a CLR response to *O*. *tsutsugamushi* infection.

## Materials and methods

### Ethics statement

UTMB complies with the USDA Animal Welfare Act (Public Law 89–544), Health Research Extension Act of 1985 (Public Law 99–158), the Public Health Service Policy on Humane Care and Use of Laboratory Animals, and NAS Guide for the Care and Use of Laboratory Animals (ISBN-13). UTMB is registered as a Research Facility under the Animal Welfare Act and has current assurance with the Office of Laboratory Animal Welfare, in compliance with NIH policy. Infections were performed following Institutional Animal Care and Use Committee approved protocols (1902006) at the University of Texas Medical Branch (UTMB) in Galveston, TX.

### Mouse infection and tissue collection

Female C57BL/6J mice were purchased Jackson Lab or Envigo RMS, Inc. Mincle^-/-^ mice on the C57BL/6J background were kindly provided by Dr. Christine Wells (University of Melbourne, Melbourne, Australia) [27]. Animals were bred and maintained under specific pathogen-free conditions. Animals were infected at 8–12 weeks of age and performed in UTMB ABSL3 facilities in the Galveston National Laboratory, and subsequent tissue processing or analysis was performed in BSL3 or BSL2 facilities, respectively. Procedures were approved by the Institutional Biosafety Committee, in accordance with Guidelines for Biosafety in Microbiological and Biomedical Laboratories. The Karp strain of *O*. *tsutsugamushi* (OtK) was utilized for all infections. Groups of 5 animals were intravenously infected with the same bacterial stock prepared from Vero cell infection, as described previously [18,28]. Mice were inoculated with a lethal dose of infection (~1.325 x 10^6^ viable bacteria, as determined via focus-forming assay) or PBS and monitored daily for weight loss and signs of disease. Lung and brain samples were collected at 2, 6, and 9–10 days post-infection with mock infected animals serving as controls and inactivated for immediate or subsequent analyses. Data shown is representative of three independent repeats.

### Infection of mouse bone marrow-derived macrophages (MΦ)

Bone marrow cells were collected from the tibia and femur of WT or Mincle^-/-^ mice and treated with red blood cell lysis buffer (Sigma Aldrich). MΦ were generated by incubating bone marrow cells at 37°C with 40 ng/ml M-CSF (Biolegend, San Diego, CA) in complete RPMI 1640 medium (Gibco), as described before [18]. Cell medium was replenished at day 3, and cells were collected at day 7. After collection and quantification, 5 x 10^5^ viable cells were seeded into 12- or 24-well plates and allowed to adhere overnight prior to infection. For TNFα-treated MΦs, recombinant mouse TNFα (Biolegend) was added 30 min prior to infection at a final concentration of 25 ng/mL [29]. Bacteria were added at a multiplicity of infection (MOI) of 2, 5, or 10 and centrifuged at 2,000 RPM for 5 min to synchronize infection. For experiments utilizing heat-killed bacteria, bacterial stocks were incubated at 56°C for 30 min [30] and used at a MOI equivalent of 10.

### Infection of mouse bone marrow-derived neutrophils

Bone marrow cells were harvested from femur and tibia of naïve mice and treated with red blood cell lysis buffer (Sigma Aldrich). Neutrophils were prepared by using anti-Ly6G magnetic beads (Miltenyi Biotec, Bergisch Gladbach, Germany); the purity of CD11b^+^Ly6G^+^ neutrophils was 96%. Cells were seeded in 24-well plates and incubated for 1 hr at 37°C with 5% CO_2_. The infection dose was 10 MOI; the Vero cell culture supernatant was used as a mock control.

### Immunofluorescence imaging

Bone marrow-derived MΦs (1x10^6^) were seeded in a 6-well plate with 22-mm round coverslips and allowed to adhere for 24 hr. Cells were then treated with 5 or 10 MOI of heat-killed or live bacteria (at indicated MOI), or 100 ng/ml of LPS for 4 or 24 hr. For experiments with TNFα, the cytokine was added 30 min prior infection at a final concentration of 25 ng/mL. Coverslips were fixed with ice cold 100% methanol and blocked with 1% BSA in PBST. Coverslips were stained with rabbit anti-OtK serum (1:1000), as described previously by our group [18,28], and rat anti-Mincle mAb (1:50, MBL International, Nagoya, Japan). Bound antibodies were visualized with Alexa Fluor 488-conjugated anti-rabbit Fab2 and Alexa Fluor 555-conjugated anti-rat IgG (1:50, clones 4412S and 4417S, respectively, Cell Signaling Technology, Danvers, MA). All samples were counterstained with DAPI (1:5000, Sigma-Aldrich). Stained coverslips were mounted on SuperFrost Plus slides (Fisherbrand) with ProLong Diamond antifade mountant (Invitrogen). Slides were imaged at the UTMB Optical Microscopy Core by using the Zeiss LSM 880 confocal microscope (405, 488, and 561 excitation lasers).

Staining of lung tissues was performed as in our previous reports [18,28]. Briefly, 8-μm frozen sections were taken from mock or lethally infected animals at day 2, 6, and 10. Sections were blocked and incubated with rabbit anti-OtK serum (1:1000) and rat anti-Mincle mAb (1:50, MBL International). Bound antibodies were visualized with Alexa Fluor 488-conjugated anti-rabbit Fab2 and Alexa Fluor 555-conjugated anti-rat IgG (1:50, clones 4412S and 4417S, respectively, Cell Signaling Technology). All samples were counterstained with DAPI (1:5000, Sigma-Aldrich). Staining with secondary antibodies and primary antibodies alone served as negative controls. Slides were imaged at the UTMB Optical Microscopy Core by using the Zeiss LSM 880 confocal microscope (405, 488, and 561 excitation lasers). Acquisition settings were identical among the experimental groups and representative images are presented from each time point.

### Nanostring gene expression profiling

Lung samples were stored in RNA*Later* (Ambion, Austin, TX) until extraction was performed. Total RNA was extracted from mock (day 0) or lethally infected lungs collected at day 2, 6, and 10, as well as mock or lethally infected brain tissues at day 10, by using the RNeasy RNA Isolation kit (Qiagen). Total RNA samples (2 mice per group, 200 ng per sample in ribonuclease-free water) were processed at the Baylor College of Medicine Genomic and RNA Expression Profiling Core (Houston, TX). Gene expression profiling was performed by using the nCounter platform and two Nanostring kits: Mouse Immunology Panel comprising 561 genes and 14 housekeeping genes; Mouse Inflammation_v2 Panel comprising 254 genes and 6 housekeeping genes (NanoString Technologies, Seattle, WA). Results from the two kits were pooled after gene expression was normalized to housekeeping gene expression and analyzed following the manufacturer’s instructions by using the nSolver Software Version 4 and Advanced Analysis Version 2.0 (NanoString Technologies).

### Quantitative reverse transcriptase PCR (qRT-PCR)

To determine host gene expression, mouse lung tissues, MΦ, and neutrophil cultures were collected in RNA*Later* or Trizol (Ambion) and incubated at 4°C overnight for inactivation. Total RNA was extracted via RNeasy mini kit (Qiagen), and cDNA was synthesized utilizing iScript cDNA kit (Bio-Rad Laboratories, Hercules, CA). qRT-PCR assays were performed using iTaq SYBR Green Supermix (Bio-Rad) on a CFX96 Touch Real-Time PCR Detection System (Bio-Rad). The assay included: denaturing at 95°C for 3 min followed with 40 cycles of: 10s at 95°C and 30s at 60°C. To check specificity of amplification, melt curve analysis was performed. Transcript abundance was calculated utilizing the 2^-ΔΔCT^ method and normalized to glyceraldehyde-4-phosphate dehydrogenase (GAPDH). Primers used in qRT-PCR analysis are listed in S1 Table.

### Bacterial load analysis (qPCR)

To determine bacterial loads, MΦ DNA were collected at 4, 24, and 48 hr after infection via a DNeasy kit (Qiagen) and used for qPCR, as described previously [16]. Bacterial loads were normalized to total nanogram (ng) of DNA per μL for each sample. Data are expressed as copy number of 47-kDa gene per ng of DNA. The copy number of the 47-kDa gene was determined by using known concentrations of a control plasmid harboring a single-copy insert of the gene. Sample gene copy numbers were then determined by a serial dilution of the control plasmid.

### Flow cytometry

Bone marrow-derived MΦ (3 x 10^6^) were cultivated as described above, aliquoted into 50 mL conical tubes (Falcon), and allowed to rest for 1 hr at 37°C. Cells were then infected with CFSE-labeled *O*. *tsutsugamushi*. CFSE-labeling was performed as previously described [31]. Briefly, CFSE (Invitrogen) was mixed with bacterial stocks at a 1:1000 ratio and incubated in dark for 10 min at 4°C. The reaction was quenched by adding complete RPMI, centrifuged at 20,000 x *g* for 10 min, and washed twice with PBS prior to addition to MΦ cultures. At 4 hr of infection, cells were collected and divided equally for surface vs. intracellular staining as previously described [18]. Cells were stained with rat anti-Mincle mAb (MBL International), AlexaFluor594-conjugated chicken-anti-rat IgG (Molecular Probes Inc, Eugene, OR), and fixed in 2% paraformaldehyde overnight at 4°C prior to analysis. Data were collected by a BD LSRFortessa (Becton Dickinson, San Jose, CA) and analyzed by using FlowJo software version 10.7.2 (Becton Dickinson).

### Western blot

Proteins from lung tissues and MΦ were extracted with RIPA lysis buffer (Cell Signaling Technology) and quantified with BCA Protein Assay kit (Thermo Fisher Scientific). Samples were stored at -80°C until processing. Thawed cell lysates were heated for 10 min at ~105°C in Laemmle buffer (Bio-Rad) containing 2-β-mercaptoethanol, loaded into 4–20% SDS-PAGE gel (Bio-Rad) then transferred onto polyvinylidene difluoride membranes (Bio-Rad). After blocking, membranes were incubated with anti-Mincle (1:500, MBL International) and anti-β-actin (1:2000, Cell Signaling Technology) and anti-rabbit/goat secondary antibodies. Pierce ECL Western Blotting substrate (Thermo Fisher Scientific) was subsequently added to the membranes and light emission was captured using Amersham Imager 680 (GE Healthcare Lifesciences, Upssala, Sweden). Quantification of band intensity was performed by using ImageJ.

### Statistical analysis

Gene expression profiling data were presented graphically as mean ± standard error of the mean (SEM) and utilized the Benjamini-Yekutieli procedure to test for significance, yielding adjusted (adj.) *p*-values. Data thereafter were analyzed using GraphPad Prism software and presented as mean ± SEM. Differences between control and treatment groups were analyzed using one-way ANOVA with Dunnett’s multiple comparisons. Statistically significant values are denoted as *p < 0.05, ** *p* < 0.01, *** *p* < 0.001, and **** *p* < 0.0001, respectively.

## Results

### Mincle is highly transcribed in murine lung tissues during terminal infection

Studies with scrub typhus animal models have revealed exaggerated, type 1-skewed immune responses in *O*. *tsutsugamushi*-infected lung tissues [16,18,28,32]; however, these studies only examined a selected panel of cytokines/chemokines. In this study, we sought to gain a broader understanding of immune crosstalk by using differential expression analysis. After infection with a lethal dose of *O*. *tsutsugamushi*, murine lungs were collected at day 2 (D2, incubation period), day 6 (D6, disease onset), and day 10 (D10, severe stage prior to host death), as described in our previous report [16]. We performed differential expression analysis on 671 immunology- and inflammation-related genes via NanoString, using mock samples (D0) as the baseline. Of note, there were no statistically significant differences by D2 for any transcriptional targets (complete list in S2 Table). Such silent responses may not be surprising given the relatively slow replication rate of *O*. *tsutsugamushi* [33], as well as our previous PCR and ELISA findings of minimal activation of cytokines/chemokines at D2 [16,18]. For D6 samples, we found 221 genes exhibiting significant changes (adj. *p* < 0.05), among which 130 genes were upregulated and 91 genes were downregulated (complete list in S3 Table). Categorically, the top-20 most highly upregulated genes were dominated by those involved in type-1 responses (*Cxcl9-11*, *Ifng*, *Il12rb1*, *Il27*), monocyte/MΦ migration and activation (*Ccl2*, *Ccl7*, *Ccl4*), as well as interferon-stimulated genes (*Socs1*, *Iigp1*, *Ifi204)*.

**Fig 1:**
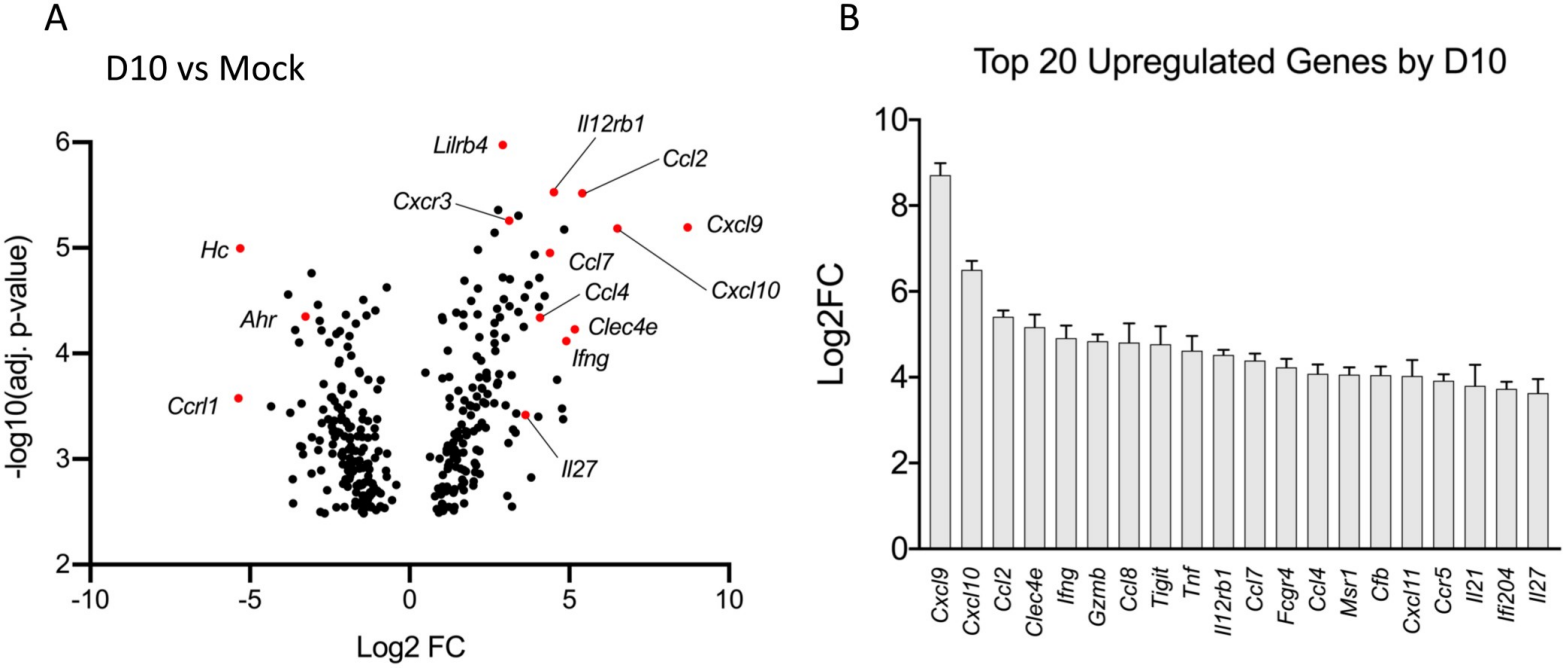
Pulmonary gene expression profiling during lethal *O*. *tsutsugamushi* infection. Nanostring gene profiling analysis was performed on RNA isolated from lung tissue homogenates of lethally infected mice at D2, D6, and D10, respectively, and compared with the mock (D0) controls (2 mice per condition). **(A)** The D10-vs-mock data, showing 312 of significantly up- or down-regulated genes (*p* < 0.05) and the markers of interest (in red). **(B)** Log2fold change (Log2FC) for the top 20 differentially upregulated genes at D10, compared to the mocks; data are shown as mean ± SEM. Differential expression analysis was performed utilizing the Benjamini-Yekutieli test for significance.

The greatest degree and spectrum of gene expression changes were found at D10 (complete list in S4 Table), with a total of 312 genes significantly differentially expressed (adj. *p* < 0.05). Of these 312 significant genes, 150 were upregulated, and 162 were downregulated (Fig 1A, with genes of interest marked in red). Fig 1B lists the top 20 upregulated genes (by fold change), including Th1- and CD8 T cell-recruiting/activating CXCR3 ligands (*Cxcl9-11*) and cytokines *(Ifng*, *Tnf*, *Il12rb1*, and *Il27)*, as well as monocyte/MΦ recruiting/activating chemokines (*Ccl2*, *Ccl7*, *Ccl4*). Notably, Mincle was the 4^th^ greatest differentially expressed gene and the only PRR observed within the top 20. Genes exhibiting the greatest downregulation included complement components (*C7*, *Vtn*, *Hc*), the atypical chemokine receptor *Ccrl1*, and the aryl hydrocarbon receptor (*Ahr*) (S1A Fig). To determine functional relationships among differentially expressed genes of significance (adj. *p* < 0.05), we queried upregulated- and downregulated-genes to the String database (string-db.org) for pathway analysis via Reactome [34]. The number of genes differentially expressed within a given Reactome pathway were ranked and plotted (S1B Fig). On D10, there was a greater degree of changes in innate immune response markers (43 genes) than adaptive immune response markers (35 genes). Overall, these findings reveal a high degree of innate immune involvement at late, rather than early (D2), stages of disease, which was concurrent with a type 1-favoring cytokine/chemokine milieu. Furthermore, the abundantly high expression of Mincle highlights the need to better understand the contribution of CLRs in innate recognition of *O*. *tsutsugamushi*.

### Mincle and signaling partners are increased in the lung and brain tissues during infection

**Fig 2:**
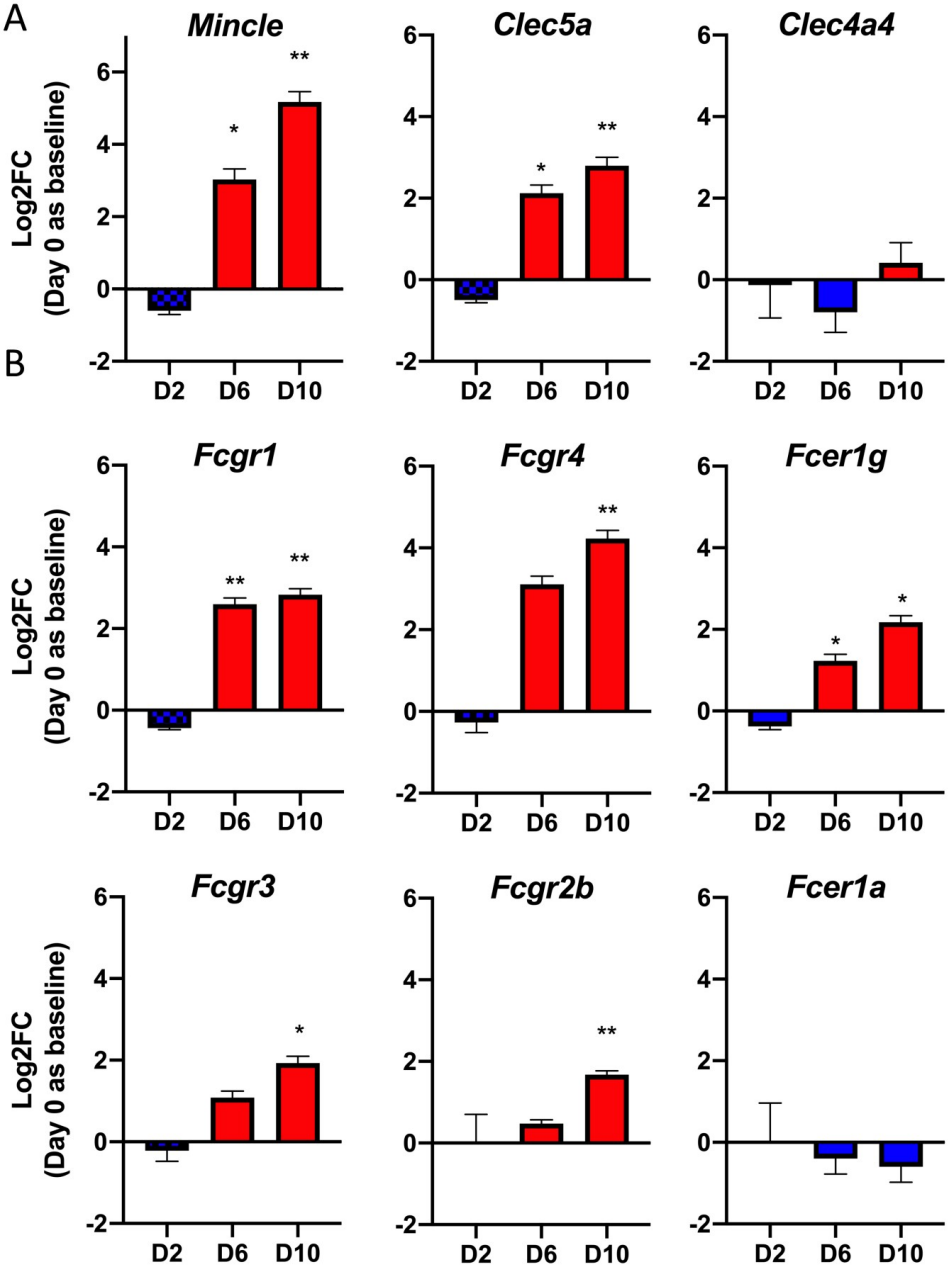
Differentially expressed CLRs and signaling partners. Nanostring gene profiling analysis was performed on lung tissue homogenates of mock and lethally infected mice, as in Fig 1. Kinetic expression data in Log2Fold change compared to the mocks for **(A)** indicated CLRs and **(B)** CLR signaling partners. Graphs are shown as mean ± SEM. Adjusted significance values for (A) and (B) were determined utilizing the Benjamini-Yekutieli test. *, *p* < 0.05; **, *p* < 0.01.

Knowledge regarding PRR recognition of *O*. *tsutsugamushi* is limited. To date, only one study revealed TLR2 activation *in-vivo* and *in-vitro* [35], while the role of other classes of PRRs has been largely unexplored [33]. Since Mincle was one of the most highly transcribed genes on D10, we examined the differential expression of other CLRs during infection. Parsing our differential expression analysis, we observed a progressive increase in Mincle and *Clec5a* transcripts. By D10, Mincle expression was increased 36-fold and *Clec5a* exhibited a 7-fold increase compared with mock controls (Fig 2A and Table 1). It is known that unlike other CLRs, Mincle and Clec5a do not possess ITAMs/ITIMs and must interact with FcγRs or *DAP10*/*DAP12*, respectively, to initiate immune responses [23]. We therefore examined whether these signaling partners were also differentially expressed during infection (Fig 2B and Table 1). As shown in Table 1, significant increases in the *Fcgr1*, *Fcgr4*, *Fcer1g*, *Fcgr3*, and *Fcgr2b* genes were consistently detected on D10, while no significant or relatively small (2-fold) increases were found for *DAP10* or *DAP12*. Likewise, D10 brain tissues had significant increases in Mincle and *Fcgr4* levels (440-517-folds), with no changes in *DAP10* or a low-level increase of *DAP12*, further highlighting the important role of Mincle/FcγR signaling in immune responses in different organs. Regarding TLR involvement in the lung, we found a relatively low, but significant upregulation of *Tlr1*, *Tlr2*, and *Tlr6* on D10, with no changes for *Tlr4*, and found a small, but significant reduction in *Tlr5* (S2 Fig). Therefore, the activation of Mincle and *Fcgr* genes in infected tissues were highly consistent and selective.

**Table 1:** Differential expression of CLRs and signaling partners in the lung and brain.

Gene	Alias/Encoded Protein	Fold Change (D10 vs. D0)	
		Lung	Brain
**Mincle**	Macrophage-Inducible C-type Lectin (*Clec4e*, *Clecsf9*)	36.00**	441.21***
***Fcgr4***	Fc-gamma Receptor 4	18.77**	517.89**
***Fcgr1***	Fc-gamma Receptor 1	7.11**	12.79*
***Clec5a***	MDL1, *Clecsf5*	6.96**	6.34*
***Fcer1g***	High affinity immunoglobulin epsilon receptor subunit gamma	4.53*	11.59*
***Fcgr3***	Fc-gamma Receptor 3	3.81*	7.93*
***Fcgr2b***	Fc-gamma Receptor 2b	3.20*	14.81
***DAP10***	*Hcst*, Hematopoietic Cell Signal Transducer	2.36	36.73
***DAP12***	*Tyrobp*, *TYRO Protein Tyrosine Kinase-Binding Protein*	2.64*	5.61*

Fold changes compared with mock controls.

*, *p* < 0.05;

**, *p* < 0.01;

***, *p* < 0.001

### Mincle predominates the CLR expression profile in infected lungs

**Fig 3:**
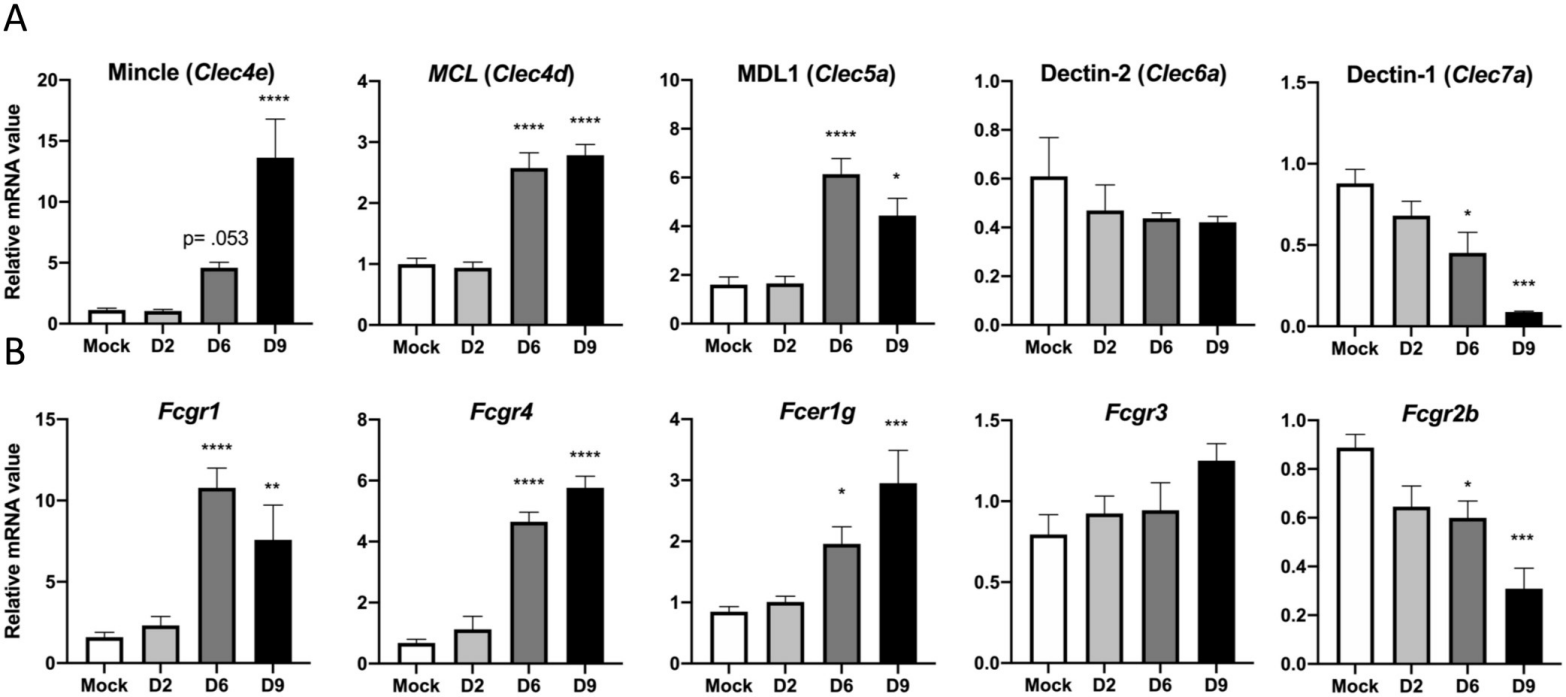
Mincle is the most predominant CLR expressed in infected lungs. qRT-PCR detection of **(A)** indicated CLRs and **(B)** *Fcgr* subtypes in whole lung tissue homogenates of lethally infected mice. Shown are representative data from 3 independent mouse infection experiments with similar trends. Data are presented relative to GAPDH values and shown as mean ± SEM. One-way ANOVA with Dunnett’s multiple comparison test was used for statistical analysis, with Mock samples used as a reference. *, *p* < 0.05; **, *p* < 0.01; ***, *p* < 0.001; ****, *p* < 0.0001.

Considering that Mincle and *Clec5a* were the only significantly changed CLRs in our gene profiling analysis, we used lung tissues for qRT-PCR analyses to validate the expression kinetics of these and other related CLRs that were not included in the NanoString kits (*Clec6a*, *Clec7a*, *Clec4b1*, *Clec9a*, *Clec12a*). We observed 13-fold upregulation of Mincle (*p* < 0.0001) and 4-fold upregulation of *Clec5a* (*p* < 0.05) at D9 (Fig 3A), corroborating our findings from differential expression analysis. We detected a 2-fold increase in *Clec12a* (*p* < 0.01), but a significant decrease in *Clec7a* (*p* < 0.001), *Clec4b1* (*p* < 0.0001), and *Clec9a* (*p* < 0.001) at D9, further suggesting that Mincle was the preeminent CLR expressed in the lungs during infection (Fig 3A and S3 Fig). Additionally, we observed a 2.5-fold upregulation of *Clec4d* (also known as MCL, *p* < 0.0001) at D9. This finding was relevant, given that Mincle and MCL belong to the asialoglycoprotein receptor family (type II), forming a heterodimeric complex which stabilizes Mincle on the cell surface [20]. This complex formation has been shown to magnify Mincle signaling through the FcγRs in the context of dendritic cells treated with trehalose-6,6’-dimycolate, the Mincle ligand from *Mycobacterium tuberculosis* [36]. Examining the *Fcgr* expression profile at D9, we observed a 7-fold upregulation of *Fcgr1* (*p* < 0.01), 5-fold upregulation of *Fcgr4* (*p* < 0.0001), and approximately 3-fold increase in *Fcer1g* (*p* < 0.001, Fig 3B). Only *Fcgr2b* exhibited a decrease in expression (*p* < 0.001), which was contrary to our differential expression findings, while *Fcgr3* exhibited no significant change. Together, our data indicate that Mincle is the most highly transcribed CLR in the lungs during *O*. *tsutsugamushi* infection, and that *Fcgr1* and *Fcgr4* (harboring activation motifs) are the likely Mincle signaling partners.

### Mincle protein levels are increased in the lungs and localized to infected regions

**Fig 4:**
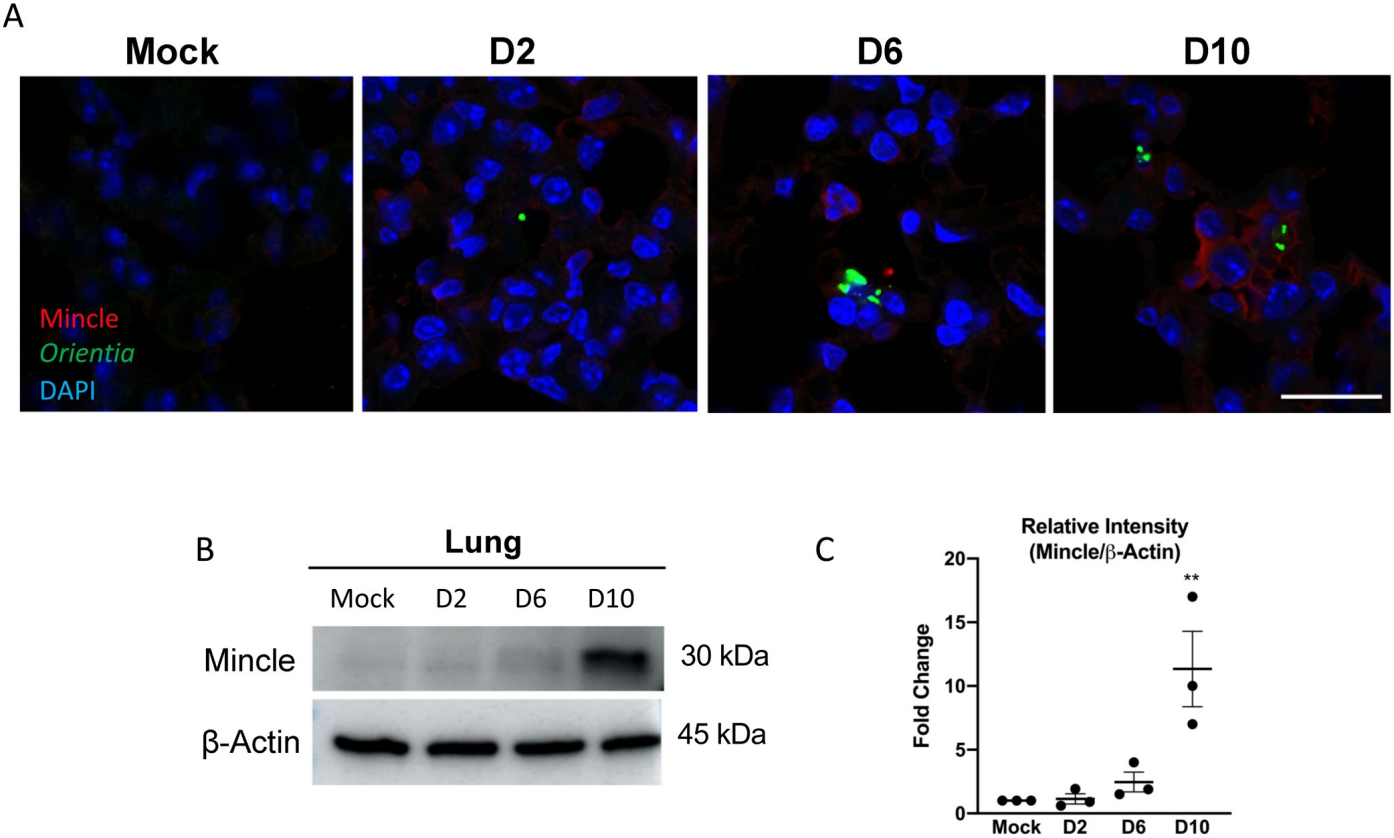
Mincle protein distribution and levels in the lungs of terminal disease. **(A)** Immunofluorescent assay detection of Mincle (red), *O*. *tsutsugamushi* (green), and DNA (DAPI, blue) in frozen lung sections prepared at the indicated days of infection. Images were taken under the same magnification (scale bar = 20 μm). **(B)** Western blots (40 μg protein/lane) of lung tissues of lethally infected mice for the levels of Mincle and β-actin. **(C)** Densitometry of representative blots from 3 independent experiments were measured via ImageJ. Fold changes were determined relative to Mincle proteins in mock samples and shown as mean ± SEM. One-way ANOVA with Dunnett’s multiple comparison test was used for statistical analysis, with mock samples used as a reference. **, *p* < 0.01.

To examine Mincle protein expression and levels in lung tissues, we performed immunofluorescent staining and Western blot. Mincle-positive (red) cells were undetectable in mock controls or weakly detected in D2 samples, but readily and intermittently detected at D6 and D10 of infection (Fig 4A). Mincle staining was observed in cells surrounding areas with detectable *O*. *tsutsugamushi* antigens (green). We found Mincle-positive cells with no bacteria, as well as bacterium-carrying cells with no or very weak Mincle staining. Western blot on whole lung tissue homogenates revealed a low, but detectable level of Mincle at D6. However, the D10 samples consistently contained the highest (~11-fold) and significantly elevated levels of Mincle (*p* < 0.01, Fig 4B and 4C). Together, these data reveal the induction of Mincle at the translational level, most prominently at the severe stages of infection.

### *O*. *tsutsugamushi* infection provokes a sustained type-1 gene activation profile in the lung

**Fig 5:**
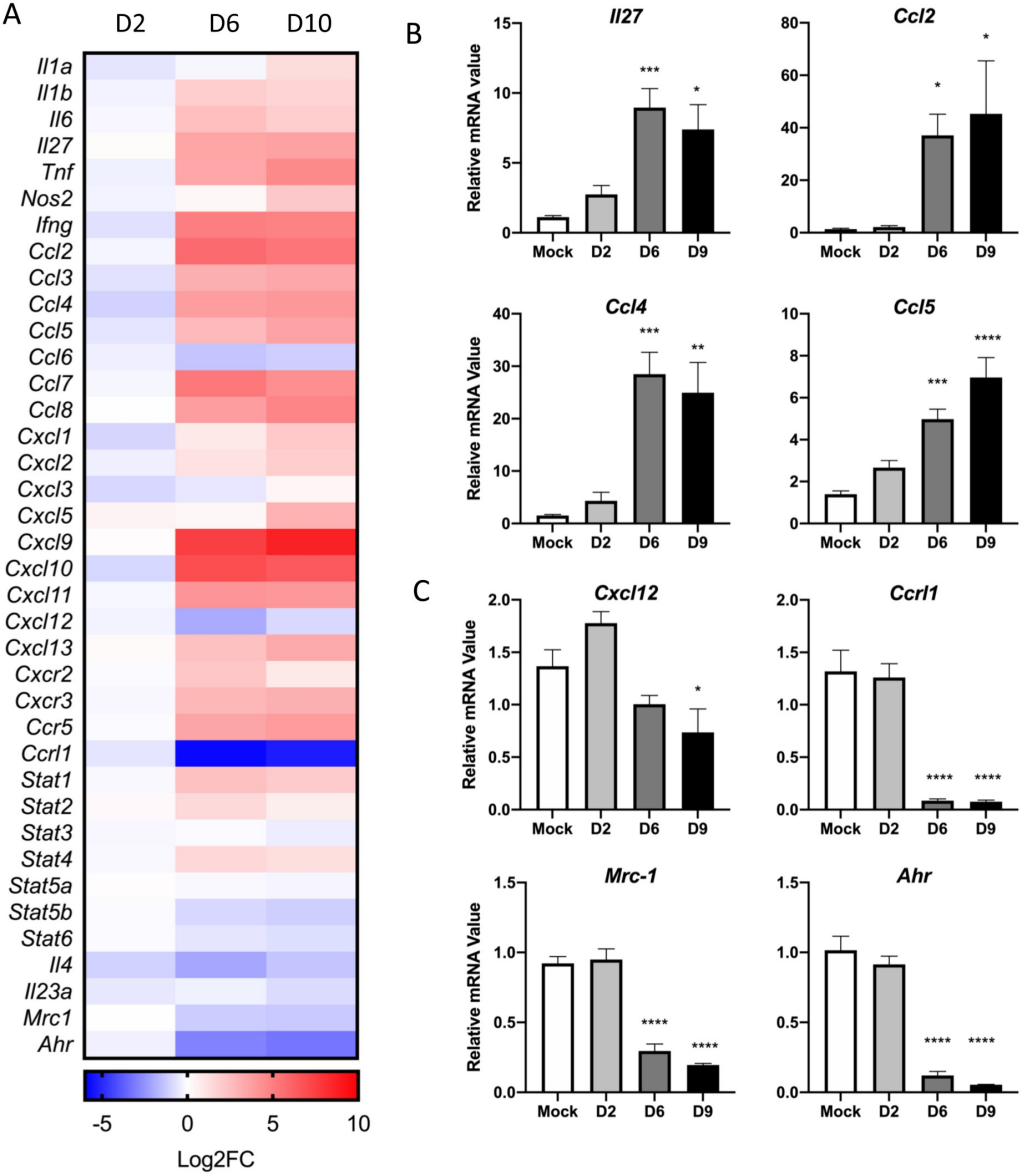
Activated type-1, but suppressed type-2, gene activation during severe disease. **(A)** Nanostring gene profiling analysis was performed on lung tissue homogenates of mock and lethally infected mice, as in Fig 1. The kinetic expression data in Log2Fold change compared to the mocks for relevant cytokines/chemokines and key inflammatory markers are shown. **(B)** Novel findings were validated by using qRT-PCR to detect type 1/proinflammatory markers, and **(C)** type 2 markers from whole lung tissue homogenates of infected mice. Shown are representative data from 3 independent mouse infection experiments with similar trends. Data are presented relative to GAPDH values and shown as mean ± SEM. One-way ANOVA with Dunnett’s multiple comparison test was used for statistical analysis, with Mock samples used as reference. *, *p* < 0.05; **, *p* < 0.01; ***, *p* < 0.001; ****, *p* < 0.0001.

Given our high levels of Mincle expression in infected tissues, we parsed our differential expression data (NanoString) and generated a heatmap distribution for the relevant immune mediators during infection (Fig 5A). At D6 and D10, we also observed significant increases in type 1/proinflammatory markers (*Il1a*, *Il1b*, *Il6*, *Il27*, *Tnf*, *Nos2*, *Ifng)*, as well as chemokines responsible for inflammatory monocyte trafficking (*Ccl2*), MΦ migration/activation (*Ccl3-5*), neutrophil trafficking (*Cxcl1*, *Cxcl2*, *Cxcl5*), and Th1 responses (*Cxcl9-11*). In contrast, there was a significant reduction or failure in activation of type 2-related chemokines/cytokines (*Cxcl12*, *Ccrl1*, *Il4*, *Il23a*) and signature markers (*Stat3*, *Stat5b*, *Stat6*, *Mrc1*, *Ahr*) during infection. These findings highlight an unbalanced proinflammatory, type 1 response to *O*. *tsutsugamushi* infection that coincides with attraction of neutrophils and monocytes through D10 of disease, corroborating our previously published studies of immune-stained lung tissues or lung-derived cells [16,18].

To validate our NanoString data, we performed qRT-PCR on infected lungs and detected a 6-fold increase in *Il27* (*p* < 0.05), 40-fold increase in *Ccl2* (*p* < 0.05), ~25-fold increase in *Ccl4* (*p* < 0.01), and 7-fold increase in *Ccl5* (*p* < 0.0001) expression at D10 (Fig 5B). These findings were in line with our previous reports for flow cytometry and ELISA studies of lung-derived immune cells [18], as these cytokine/chemokines are known to be associated with Th1 and M1 MΦ polarization [37]. Concurrently, a significant reduction in type 2- or tissue healing-related genes, including *Cxcl12* (*p* < 0.05), *Ccrl1* (*p* < 0.0001), *Mrc-1* (*p* < 0.0001), and *Ahr* (*p* < 0.0001), provided additional evidence for skewed type 1, but absent type 2, immune responses in our model of severe scrub typhus (Fig 5C).

### MΦ upregulation of Mincle and proinflammatory markers is dependent on *O*. *tsutsugamushi* doses and replication

**Fig 6:**
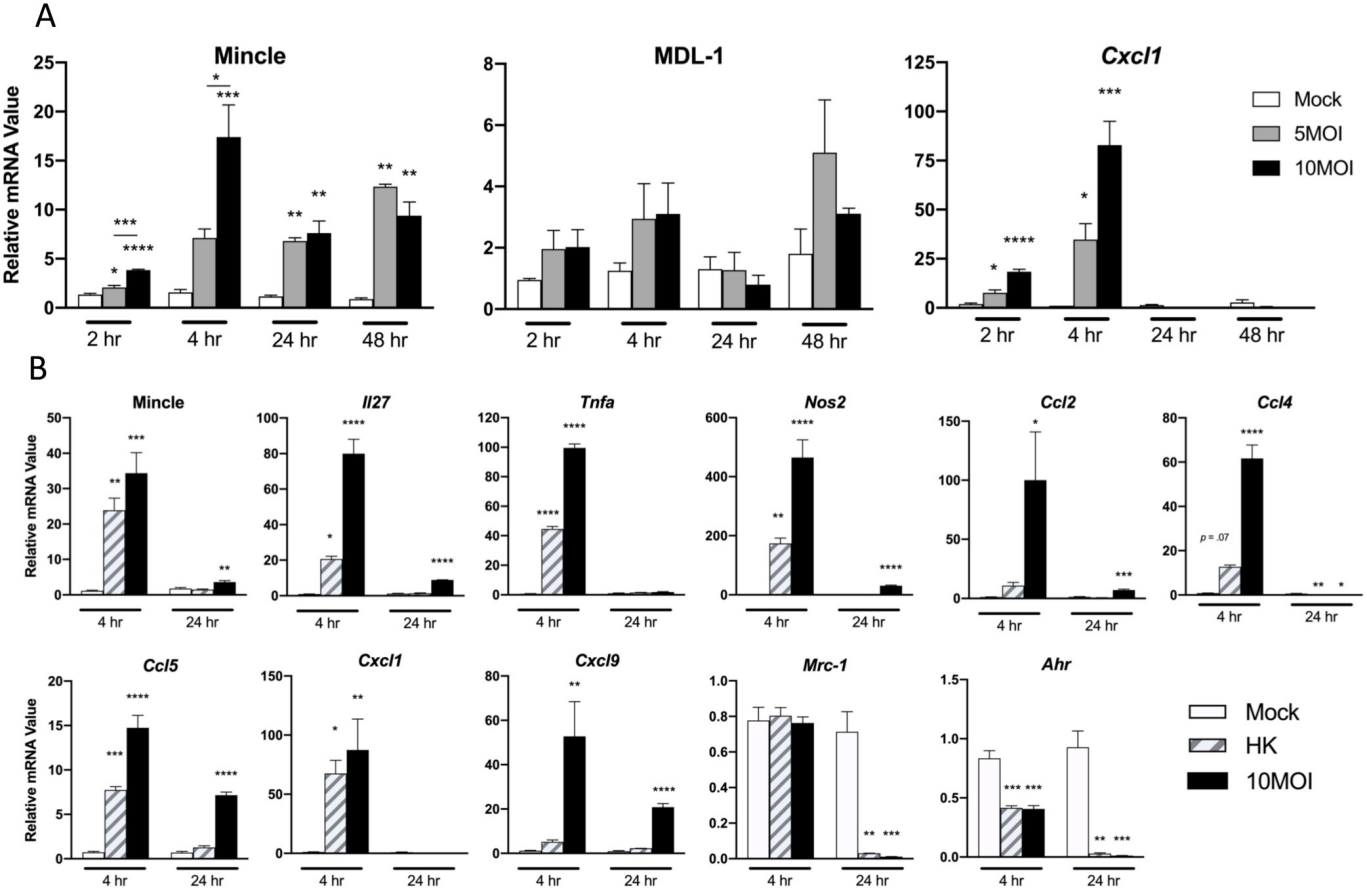
Dose-dependent and sustained activation of Mincle and type-1 markers in MΦ exposed to *O*. *tsutsugamushi*. Bone marrow-derived MΦ of WT C57BL/6J mice were exposed to live bacteria (MOI 5 or 10) or heat-killed bacteria (HK, MOI 10 equivalent). **(A)** qRT-PCR analyses of indicated genes at 2 to 48 hr post-infection. **(B)** qRT-PCR analyses of indicated genes at 4 and 24 hr of incubation with live vs. HK bacteria. Data are presented relative to GAPDH and shown as mean ± SEM. One-way ANOVA with Dunnett’s multiple comparison test was performed, using uninfected controls for each time point as the reference. *, *p* < 0.05; **, *p* < 0.01; ***, *p* < 0.001; ****, *p* < 0.0001.

We have reported that sustained recruitment and activation of M1-like MΦs and neutrophils in inflamed lung tissues is a hallmark in murine models of lethal scrub typhus [18,28]. Since Mincle may be expressed in both MΦs and neutrophils, we sought to examine the cellular sources of Mincle expression during *O*. *tsutsugamushi* infection. We first evaluated the Mincle transcription profile in infected neutrophils (MOI 10) and observed a small (~2-fold), but significant (*p* < 0.01), increase in Mincle transcription at 4 hr, which was completely diminished by 18 hr (S4 Fig). We then infected bone marrow-derived MΦs with *O*. *tsutsugamushi* (MOI 5 or 10) and examined a panel of CLR and cytokine/chemokine transcripts at 2 to 48 hr of infection. Mincle transcription was significantly increased throughout the course of infection, with a peak increase of 17-fold (*p* < 0.001) at 4 hr for the high-dose group, but a peak increase of 12-fold (*p* < 0.01) at 48 hr for the low-dose group (Fig 6A). This infectious dose-dependent increase of Mincle, but not MDL-1, at 2 and 4 hr suggested a selective induction of Mincle by *O*. *tsutsugamushi*. It is known that *Cxcl1*, a neutrophil chemoattractant, is induced by Mincle activation [38]. Indeed, we found an infectious dose-dependent increase of *Cxcl1* starting at 2 hr and peaking at 4 hr (80-fold increase in the high-dose group, *p* < 0.001). Together, our data indicate a selective upregulation of Mincle in response to *O*. *tsutsugamushi* infection that is most prominent in MΦs.

The early induction of Mincle in response to live bacteria inspired us to further examine MΦ responses to heat-killed (HK) bacteria at a concentration equivalent of 10 MOI. We focused on two life cycle stages of *O*. *tsutsugamushi*: at 4 hr (when bacteria have just escaped from the endosome/phagosome to inhabit the cytoplasm) and at 24 hr (when bacteria are located near the perinuclear region for initial replication) [11,33]. While both inactivated and live bacteria induced a similar degree of Mincle upregulation at 4 hr, only live bacteria generated a sustained response at 24 hr (Fig 6B). With regard to inflammatory responses to HK and live bacteria, we observed only live *O*. *tsutsugamushi* generated significant and sustained expression of type 1-promoting cytokines/chemokines *Il27*, *Nos2* (M1 MΦ marker), *Ccl2* and *Ccl5* (proinflammatory, M1-skewing chemokines), as well as *Cxcl9*. Concurrently, there was a significant reduction in *Ahr* and *Mrc-1* (M2 activation markers) following exposure to live and inactivated bacteria. These results suggest both bacterial growth-dependent and -independent activation of Mincle and its related inflammatory responses in MΦs.

Having documented Mincle upregulation during *O*. *tsutsugamushi* infection MΦs, we then examined whether Mincle contributes to the generation of proinflammatory responses. We used WT or Mincle^-/-^ MΦs to measure the levels of Mincle and a panel of cytokines/chemokines during infection with different doses of live bacteria (5 MOI or 10 MOI). As shown in (S5A Fig), there was an infectious dose-dependent increase in Mincle transcription in WT cells at 4 hr post-infection, but not in Mincle^-/-^ cells. While there was an infectious dose-dependent increase in *Cxcl1*, *Ccl2*, *Tnfa*, and *Il27* in WT cells, Mincle^-/-^ cells treated with a high-dose of *O*. *tsutsugamushi* exhibited abrogated transcription of *Cxcl1* (*p <* 0.05) and *Ccl2* (*p* = 0.06). Mincle^-/-^ cells also produced less *Nos2*, *Tnfa*, *Il27*, and *Cxcl9* than WT cells upon infection, but the reduction did not meet statistical significance under the tested infection doses and time. At 24 hr post-infection, we observed significant increases in WT production of *Il27*, *Cxcl10*, and *Cxcl9*, which were diminished in Mincle^-/-^ cells (S5B Fig). Nevertheless, we found comparable *O*. *tsutsugamushi* copy numbers in Mincle^-/-^ and WT MΦ at 4, 24, and 48 hr after infection at 2 MOI or 5 MOI, respectively (S5C Fig). Therefore, Mincle gene deletion does not alter bacterial entry or replication in vitro, allowing us to focus on examining the role of Mincle in host inflammatory responses.

### Mincle protein expression increases in MΦs exposed to *O*. *tsutsugamushi*

**Fig 7:**
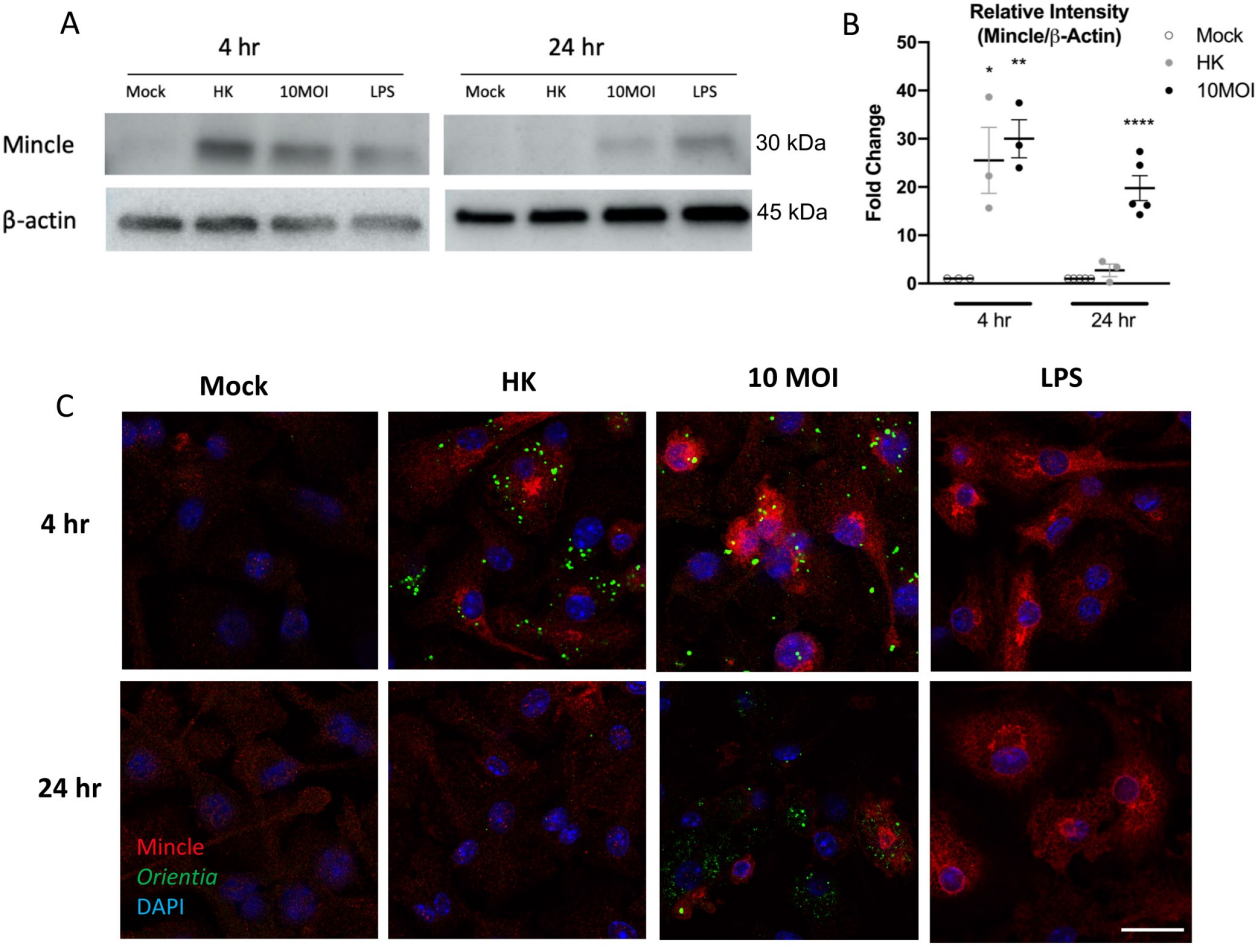
Mincle levels and distribution in MΦ exposed to live and inactivated bacteria. Bone marrow-derived MΦ of C57BL/6J mice were exposed to live bacteria (MOI 10), heat-killed bacteria (HK, MOI 10 equivalent), or LPS (100 ng/ml, as a positive control for Mincle expression). **(A)** Western blots (5 μg protein/lane) of cell lysates for the levels of Mincle and β-actin at 4 and 24 hr. **(B)** Densitometry of bacterium-exposed groups with a minimum of 3 representative blots by using ImageJ. Fold changes were determined relative to Mincle proteins in control samples and shown as mean ± SEM. **(C)** Immunofluorescent assay detection of Mincle (red), *O*. *tsutsugamushi* (green), and DNA (DAPI, blue) in cells at 4 and 24 hr of incubation (scale bar = 20 μm). One-way ANOVA with Dunnett’s multiple comparison test was used for statistical analysis, with mock samples as the references. *, *p* < 0.05; **, *p* < 0.01; ****, *p* < 0.0001.

To confirm Mincle protein levels in infected MΦs, we performed western blot and included LPS stimulation as a positive control. We found a marked increase in Mincle protein levels at 4 hr post-exposure to live or HK bacteria; however, at 24 hr, Mincle proteins were detected only in live bacterium-exposed MΦs (Fig 7A and 7B), which were consistent with our PCR findings (Fig 6). We then examined the Mincle expression pattern in MΦ exposed to live or HK bacteria to determine whether signal colocalization would occur. Indirect immunofluorescent staining and confocal image analyses revealed comparable Mincle positivity (red) at 4 hr post-exposure to live or HK bacteria (green), on par with LPS stimulation (Fig 7C). While Mincle-positive staining was detected with or close to *O*. *tsutsugamushi* antigen-positive cells, there was limited evidence of Mincle-bacterium colocalization. At 24 hr, while Mincle staining was still readily detectable in MΦs exposed to live bacteria, only baseline levels of staining were found in MΦs exposed to HK bacteria. Collectively, our data indicate that *O*. *tsutsugamushi* antigens are capable of stimulating Mincle protein expression in MΦs, with the most pronounced response evident at early, rather than late, time points.

### *O*. *tsutsugamushi* stimulates Mincle expression in both infected and uninfected MΦs

**Fig 8:**
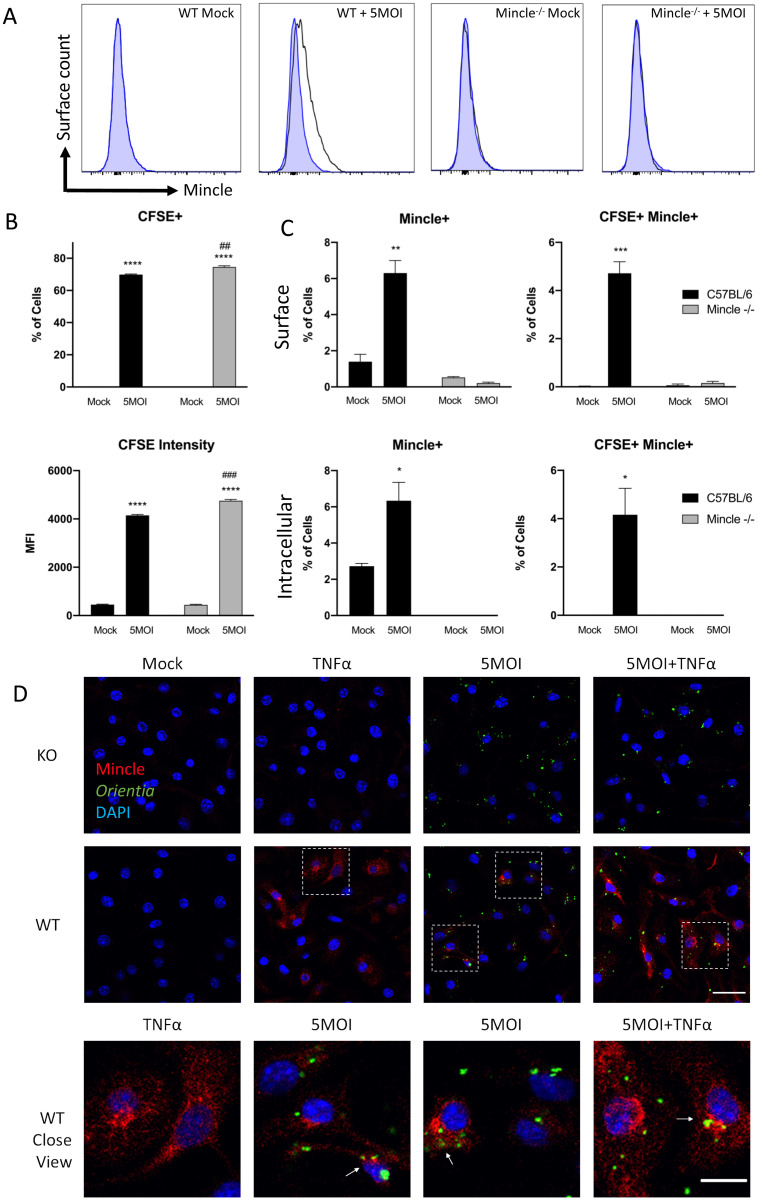
Mincle-expressing MΦ are increased during infection and promoted by TNFα. Bone marrow-derived WT or Mincle^-/-^ MΦ were exposed to *O*. *tsutsugamushi* (5 MOI) and analyzed via flow cytometry and IFA at 4 hr post-infection. **(A)** Representative frequencies of Mincle^+^ cells detected for surface staining, with WT and Mincle^-/-^ treatment groups compared with WT mock samples (blue). **(B)** The percentage and mean fluorescent intensity (MFI) of CFSE^+^ cells are shown for intracellularly stained MΦs among bacterium-carry (CFSE^+^) cells. **(C)** A comparison of surface vs. intracellular Mincle staining, showing percentages of Mincle^+^ and CSFE^+^Mincle^+^ cells. **(D)** Immunofluorescent assay detection of Mincle (red), *O*. *tsutsugamushi* (green), and DNA (DAPI, blue) in cells pretreated with TNFα (25 ng/mL for 30 min) prior to infection (scale bar = 10 μm for close-view images, 20 μm for all others). Unpaired t-test was used for statistical analysis. For comparison with mock controls within WT or Mincle^-/-^ cells *, *p* < 0.05; **, *p* < 0.01; ****, *p* < 0.0001. For comparison between WT and Mincle^-/-^ cells ##, *p* < 0.01, ###, *p* < 0.001.

Since our IFA findings revealed Mincle expression in both infected and uninfected MΦs, we sought to measure Mincle protein levels via using flow cytometric analysis. First, we observed increased frequency of Mincle^+^ cells in WT MΦ at 4 hr after infection (5 MOI), with no changes in Mincle^-/-^ cells (Fig 8A), corroborating our IFA findings (Fig 7). Then, we utilized CFSE-labeled *O*. *tsutsugamushi* to categorize infected (CFSE^+^) or uninfected (CFSE^-^) cells at 4 hr. We found that 57% of WT MΦ were infected (*p* < 0.0001), while 64% percent of Mincle^-/-^ cells were infected (*p* < 0.0001) (Fig 8B). Though small, the difference in percent infected Mincle^-/-^ cells and WT cells was significant (*p* < 0.05). The mean fluorescent intensity (MFI) of CFSE was also significantly (*p* < 0.001) greater in Mincle^-/-^ cells when compared with WT. Additionally, our analysis revealed that 6% percent of WT cells were Mincle^+^ (*p* < 0.01) and 4.5% of cells were CFSE^+^Mincle^+^ (*p* < 0.001) after infection, with comparable results between surface stained and permeabilized MΦ (Fig 8C). Together, our data indicate both infected and uninfected cells express Mincle.

To examine whether and how host factors contribute to the Mincle expression, we focused our studies on TNFα, because it is known to induce Mincle in murine MΦ [39] and is highly upregulated in lungs of lethally infected mice (Fig 1) [16]. Cells were primed with TNFα for 30 min before the infection. At 4 hr post-infection, we detected increased Mincle expression (red) in WT cells receiving TNFα or *O*. *tsutsugamushi* (5 MOI) alone, with more intense staining observed in cells receiving TNFα 30 min prior to infection (Fig 8D). Though Mincle staining was evident near *O*. *tsutsugamushi* antigens (green), we observed limited colocalization (arrows), consistent with our result shown in Fig 7. Therefore, our FACS and IFA results are consistent, implying a role of TNFα in inducing Mincle and a potential interplay between *O*. *tsutsugamushi*- and TNFα-mediated Mincle responses.

### TNFα amplifies Mincle-dependent inflammation in MΦs exposed to *O*. *tsutsugamushi*

**Fig 9:**
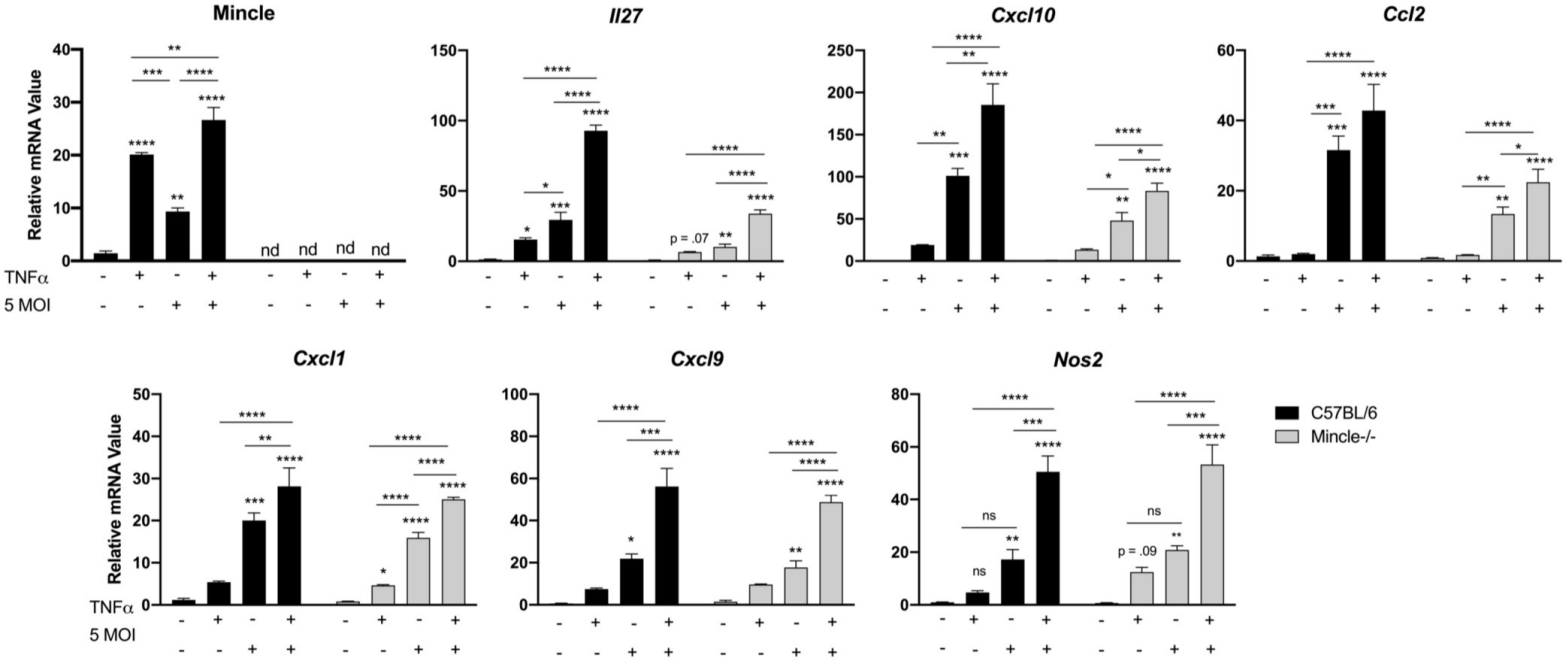
Effect of TNFα on Mincle transcription and exacerbation of Mincle-dependent inflammatory responses in infected MΦs. Bone marrow-derived wild-type (WT) or Mincle^-/-^ MΦ were primed with TNFα (25 ng/ml for 30 min) prior to infection with live bacteria (5 MOI). qRT-PCR analyses of indicated genes (relative to GAPDH) at 4 hr post-infection are presented; data are shown as mean ± SEM. One-way ANOVA with Dunnett’s multiple comparison test was performed for treatment groups within the WT or Mincle^-/-^ MΦ backgrounds, respectively. *, *p* < 0.05; **, *p* < 0.01; ***, *p* < 0.001; ****, *p* < 0.0001.

Based on the observation that MΦs treated with TNFα prior to *O*. *tsutsugamushi* infection exhibited pronounced Mincle staining intensity, we asked whether TNFα may modulate Mincle-related inflammation. Using qRT-PCR, we found that WT MΦ treated with TNFα 30 min prior to infection with *O*. *tsutsugamushi* (5 MOI) exhibited significantly greater Mincle expression (25-fold increase, *p* < 0.0001) at 4 hr after infection than cells receiving TNFα (20-fold increase, p < 0.001) or *O*. *tsutsugamushi* alone (8-fold increase, *p* < 0.01, Fig 9). We also observed similar trends for the key proinflammatory markers *Cxcl1*, *Nos2*, *Il27*, *Cxcl9*, and *Cxcl10*. Overall, Mincle^-/-^ MΦ presented parallel trends for the inflammatory markers analyzed. However, we observed that the degree of *Il27*, *Ccl2*, and *Cxcl10* transcripts generated in infected Mincle^-/-^ cells receiving TNFα pretreatment was less than half that of their WT comparators. The differences in *Il27* and *Cxcl10* production in infected Mincle^-/-^ and WT cells was even more pronounced by 24 hr post-infection (S6 Fig). Together, our data reveal that TNFα may promote and exacerbate Mincle-mediated inflammation.

## Discussion

Despite being an important emerging infectious disease, detailed immunological studies of scrub typhus patient samples or animal tissues are scarce. This study was aimed at understanding the drivers of type 1-skewed immune responses and exploring potential PRRs sensing *O*. *tsutsugamushi*. Through differential expression and immunological analyses of lung tissues from lethally infected mice, as well as *in vitro* infection of murine primary MΦs with viable and inactivated *O*. *tsutsugamushi*, we revealed the upregulation of Mincle at the transcriptional and translational levels. Additionally, we found abrogated proinflammatory responses in Mincle^-/-^ cells in responding to *O*. *tsutsugamushi* infection in the presence or absence of exogenous TNF-α treatment. This study is important in several aspects, suggesting that *O*. *tsutsugamushi* can stimulate Mincle and promote type 1-skewed proinflammatory immune responses in host cells.

Firstly, the NanoString study described herein offered comprehensive profiles of important host immune response/inflammation genes in the lungs at different stages of infection. This data confirmed and greatly expanded previous animal studies that examined a limited number of cytokine/chemokine genes in visceral organs via PCR during *O*. *tsutsugamushi* infection [16,28,32]. Our finding of *Il27* upregulation in inflamed lung tissues (Fig 1) was important, given our previous study revealing increased IL-27 protein via Bioplex in infected lungs [17]. The identification of *Il27* and other immunologic pathways that were significantly increased (*Ccl2-5*) or reduced (*Ccrl1*, *Ahr*) during infection have opened new avenues of investigating immune responses to *O*. *tsutsugamushi*.

Secondly, the PRR activation profile to *O*. *tsutsugamushi* is scarcely understood; our studies with mouse tissues and cultured MΦs has suggested an important role for CLRs, but not TLRs, in response to this Gram-negative bacterium. One study has described TLR2 activation in *O*. *tsutsugamushi*-infected mice and cultured HEK293 cells [35] and others identified no role for TLR4 in mice during infection [40,41]. These reports were consistent with our observation of low TLR2 and no TLR4 induction *in vivo* (S2 Fig). For intracellular bacteria replicating in the cytosol, activation of cytoplasmic DNA sensors, such as STING and RIG-I, would likely occur; however, evidence for the activation of these sensors remains speculative [41]. Our discovery of two highly upregulated CLRs, Mincle and *Clec5a*, in lethally infected mice was novel and significant. Among these two CLRs, Mincle was likely the most important sensor for *O*. *tsutsugamushi*, because 1) it was the 4^th^ most upregulated gene in the lungs by the terminal phase of disease and the only PRR within the top-20 (Fig 1); 2) the timing and peak activation of Mincle coincided with the onset of disease and progression of lethal infection in the lungs (Figs 2 and 3) and brains (Table 1) of mice; and 3) signaling partners for Mincle (*Fcgr1* and *Fcgr4*), but not those for Clec5a (*DAP10* or *DAP12*), displayed a high degree of upregulation, implicating Mincle pathways as potentially playing a more compelling role during infection (Figs 2 and 3). This conclusion was further supported by the positive correlation between the peak upregulation of proinflammatory cytokines/chemokines through the terminal phase of disease (Fig 5).

At present, Mincle has mainly been characterized in the context of MΦ-pathogen interactions, especially for M1 polarization and proinflammatory responses to *M*. *tuberculosis* [25,42]. MΦs play key roles in infection with *O*. *tsutsugamushi* and other closely related *Rickettsia* species [43–46]. This study, for the first time, revealed *O*. *tsutsugamushi* infection-triggered Mincle expression in M1-like MΦs in an infectious dose-dependent manner (Fig 6). We were surprised at the capacity of inactivated *O*. *tsutsugamushi* to stimulate transient Mincle expression along with other proinflammatory or M1-promoting genes (*Il27*, *Nos2*, *Tnf*, *Ccl5*, *Cxcl1*), but not *Ccl2*, *Ccl4*, and *Cxcl9* at 4 hr, while strong or significant upregulation was sustained in live bacteria-exposed cells at 24 hr. Mincle^-/-^ MΦ infected with live bacteria produced significantly less *Cxcl1* and *Ccl2* early in infection than WT cells (S5A Fig), indicating this receptor plays a role in propagating inflammation. Since Mincle^-/-^ and WT MΦ exhibited comparable bacterial loads from 4–48 hr of infection *in vitro* (S5C Fig), we speculate that Mincle enhances proinflammation and type 1-mediated response in target cells and inflamed tissues, rather than directly influences *O*. *tsutsugamushi* invasion and intracellular replication in target cells.

Nevertheless, our cytokine signatures for WT cells largely agree with previous studies that explored cytokine profiles of human monocytes/MΦ responding to live *O*. *tsutsugamushi* infection [14,47]. Our findings are also relevant to the basic biology of *O*. *tsutsugamushi*, as the early phase (4 hr) represents the stage immediately after endosomal/phagosomal escape, whereas the later phase (24 hr) coincides with the start of bacterial replication [33]. The reduction in the magnitude of immune responses at 24 hr of infection (Fig 6) may be due to the activation of cellular regulatory molecules/pathways, or pathogen-driven mechanisms, as *O*. *tsutsugamushi* can actively modulate host immune responses during infection. Since *O*. *tsutsugamushi* bacteria replicate very slowly (with ~10-hr doubling time in vitro)[48, 49], the capacity of evading host immune responses is critical for their successful replication. For example, *O*. *tsutsugamushi* can actively thwart the NF-κB activation pathway by 24 hr of infection [29,50]. Regardless of the underlying mechanisms, our findings of rapid Mincle expression elicited by inactivated bacteria suggest the presence of potential bacterial ligands for Mincle recognition, which were preserved in our heat-inactivation procedure. This is important as it may allow HK bacteria to be explored further for the identification of putative Mincle ligands, precluding the technical and logistical challenges associated with performing such experiments in BSL-3 containment facilities.

Thirdly, we revealed a unique Mincle protein expression pattern in infected lung tissues and MΦs (Figs 4, 7, and 8). Looking at the cellular expression profile for Mincle in the lungs, we observed Mincle staining in cells nearby *O*. *tsutsugamushi* antigen by D10 (Fig 4). This suggested that non-infected cells may be responding to bacterial components, or alternatively, to cytokines/chemokines. While we did not perform co-staining for specific cell phenotypes, we speculated that Mincle-positive cells were most likely infiltrating monocytes/MΦs, based on our previous FACS analyses of lung-derived cells [18]. Our *in-vitro* studies and IFA staining of infected MΦ supported this notion and revealed an intriguing pattern of Mincle expression. Early in infection, comparable levels and patterns of Mincle proteins were detected in cells treated with either inactivated or live bacteria (Fig 7). Later in infection (24 hr), however, only cells infected with viable bacteria had detectable Mincle, though overall signal intensity was lower compared with the early timepoint. These IFA findings were in congruence with our qRT-PCR and WB data, revealing the importance of viable bacteria in generating sustained Mincle expression. The IFA findings of Mincle positivity in both bystander and infected cells after treatment with live or inactivated *O*. *tsutsugamushi* suggested to us that bacterial components may not be the sole source underlying our findings. This notion was further supported by our flow cytometric data, confirming that both infected and uninfected cells can express Mincle following exposure to live *O*. *tsutsugamushi* (Fig 8). Of note, though Mincle is is known to be anchored to the cytoplasmic membrane, we observed a diffuse cytoplasmic staining pattern in our infected or LPS-stimulated MΦs (Fig 7), which were consistent with other reported IFA studies in MΦ-like RAW264.7 cells stimulated with LPS *in-vitro* [51] and in dermal dendritic cells *in situ* [52]. To confirm an intracellular pool of Mincle proteins, we analyzed the percent Mincle^+^ cells from surface vs. intracellular stained MΦ via flow cytometry. We confirmed that while the majority of Mincle proteins were detected via surface staining, the percent Mincle^+^ cells determined via intracellular staining was slightly greater (Fig 8). While it remains unclear as to whether the minimal co-localization of Mincle and Oriential antigens in infected lung tissues and MΦs was due to the unique biology of *O*. *tsutsugamushi* or technical issues related to antibodies used in this study, it was evident that Mincle is not required for *O*. *tsutsugamushi* entry or replication (Fig 8B and S5 Fig).

Finally, Mincle expression and related inflammation were likely initiated via multiple avenues during *O*. *tsutsugamushi* infection, including via bacteria-related components, cytokine/chemokine production, and host DAMP release. This study confirms a previous report describing TNFα as a strong stimulus of Mincle expression [39]. Importantly, we revealed a significant, TNFα-mediated amplification of Mincle expression, as well as proinflammatory expression (*Il27*, *Cxcl9*, *Cxcl10*, *Ccl2*, *Cxcl1*, *Nos2*) in the context of *O*. *tsutsugamushi* infection (Fig 9). More importantly, we found that such TNFα-mediated enhancement during infection was 2- to 3-folds reduced in Mincle^-/-^ MΦs (*Il27*, *Cxcl10*, *Ccl2*), implying the mitigation of proinflammatory responses. Our data collectively indicate a prominent role of Mincle in magnifying inflammatory response in a TNFα-rich environment. This finding has important implications, since TNFα levels are known to correlate of disease severity in human scrub typhus [53], and that TNFα is highly upregulated in the lungs of lethally infected mice (Fig 1) [16]. Studies are ongoing to examine possible mitigation of proinflammation and acute tissue injury during infection in Mincle^-/-^ mice (plus anti-TNFα treatment). Future investigation should also be aimed at defining potential bacterial components that could induce Mincle expression. As an obligately intracellular pathogen, *O*. *tsutsugamushi* relies on the host cell as a source of nutrients [33]. Therefore, it is conceivable that enzymatic altering of host cell proteins or lipids during this process may, in turn, stimulate Mincle, in a similar manner to that described for *Helicobacter pylori* [26]. Identification of such a ligand, however, presents major challenges due to the lack of available tools for genetic manipulation of *O*. *tsutsugamushi* at the present time [7,11].

**Fig 10:**
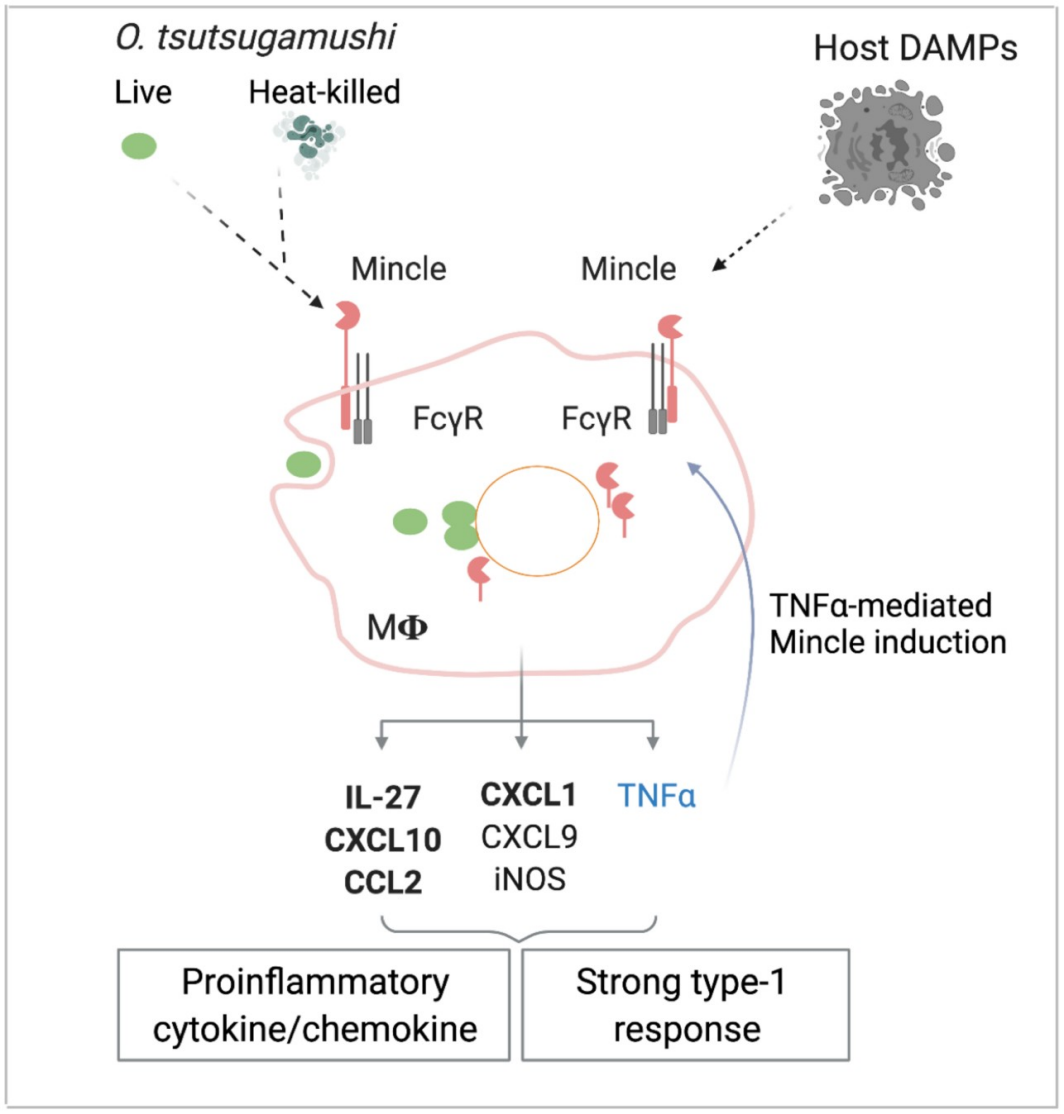
A schematical model for the role of Mincle/FcγR in MΦ responses to *O*. *tsutsugamushi*. *O*. *tsutsugamushi* bacteria and yet-undefined bacterial components, as well as damaged host molecules (DAMPs) can selectively stimulate Mincle/FcγR expression in MΦ, leading to subsequent production of proinflammatory cytokines/chemokines in inflamed tissues. While such immune responses help the control of bacterial replication, sustained Mincle-related responses also activate Mincle-dependent proinflammatory factors, including IL-27, CXCL10, CXCL1, and CCL2 (in bold). Meanwhile, Mincle-independent host factors (e.g., TNFα) can further promote the magnitude of Mincle expression via a positive regulation loop. Collectively, these events promote type 1-skewing immune responses, cellular damage, and acute lung injury.

Based on our findings presented herein, we propose a hypothetic model for the contribution of Mincle and related pathways in immune responses to *O*. *tsutsugamushi* infection (Fig 10). MΦs can sense invading or inactivated bacteria via inducing Mincle and FcγR expression at the initial stages of infection. MΦs can also respond to released host DAMPs and TNFα, as intracellular bacterial growth progresses, via increased Mincle expression on the cell surface and in an intracellular pool. The activation of Mincle/FcγR promotes the expression of diverse proinflammatory cytokines/chemokines for potential recruitment and activation of phagocytes (CXCL1, CCL2, TNFα, iNOS), as well as Th1 and CD8^+^ T cells (CXCL9, CXCL10, IL27, TNFα). These M1-like MΦs help control bacterial infection; however, excessive TNFα in the microenvironment can result in sustained Mincle expression and MΦ inflammatory responses. These innate responses may ultimately contribute to Th1/CD8-skewed responses and acute tissue damage, as we observed in the lungs and other organs [17,18,54]. This model suggests that besides Mincle^-/-^ mice, future investigation with targeted blockage of TNFα- or FcγR1/FcγR4-mediated pathways will help define the interplay of TNFα/Mincle/FcγR signaling in the regulation of host-*O*. *tsutsugamushi* interactions and pathology of scrub typhus. Such studies would also help understand immune responses against other obligately intracellular pathogens, including *Rickettsia*, *Anaplasma*, *Ehrlichia*, and *Chlamydia*, for which virtually no information is yet available regarding the role of Mincle or other CLRs in pathogen recognition.

In summary, this study revealed new insights into the innate immune recognition of *O*. *tsutsugamushi*. Through comprehensive differential expression analysis of mouse lung and brain tissues, we provided the first evidence of Mincle involvement during *O*. *tsutsugamushi* infection. Our observations from *in-vitro* MΦ infection suggest a selective upregulation of Mincle that may rely on bacterial growth-dependent and -independent factors, as well as TNFα stimulation. To-date, CLRs represent a virtually unexplored realm of immunology for *O*. *tsutsugamushi* and other obligately intracellular bacteria. While potential bacterium- and/or host-derived ligands for Mincle/FcγR signaling remain unknown, it is conceivable that Mincle activation during infection may contribute to or drive an overzealous type 1 response in both innate and adaptive immune cells, leading to progressive tissue damage during *O*. *tsutsugamushi* infection. A better understanding of how Mincle activation contributes to immune dysregulation may aid the design of treatments for severe scrub typhus.

## Supporting information

S1 Table Primer sequences for qRT-PCR analysis used within the study. (DOCX)

S2 Table Complete list of differentially expressed genes in lung tissues (D2 vs. mocks). (DOCX)

S3 Table Complete list of differentially expressed genes in lung tissues (D6 vs. mocks). (DOCX)

S4 Table Complete list of differentially expressed genes in lung tissues (D10 vs. mocks). (DOCX)

S1 Fig Pulmonary gene expression profiling and functional pathway analysis during lethal *O*. *tsutsugamushi* infection. Nanostring gene profiling analysis was performed on RNA isolated from lung tissue homogenates of lethally infected mice at D10 and compared with the mock controls. **(A)** Shown are the bottom 20 most downregulated genes by Log2FC. **(B)** Significantly upregulated or downregulated genes were input to the String database and analyzed by the Reactome pathway. Graphs are shown as mean ± SEM. Differential expression analysis was performed utilizing the Benjamini-Yekutieli test for significance.
(TIF)

S2 Fig Differential expression analyses of Toll-like receptors in the lung of lethally infected mice. Nanostring gene profiling analysis was performed on RNA isolated from lung tissue homogenates of lethally infected mice at D2, D6, and D10, respectively, and compared with the mock controls. We then parsed our data to examine expression of *Tlr* genes. Scale is Log2Fold change compared to mock. Graphs are shown as mean ± SEM. Significance was determined utilizing the Benjamini-Yekutieli test. *, *p* < 0.05, ** *p* < 0.01.
(TIF)

S3 Fig Expression of additional CLRs in the lungs of lethally infected mice. Whole lung tissue homogenates from lethally infected mice were measured for expression of indicated CLRs via qRT-PCR. All data are presented relative to GAPDH values and shown as mean ± SEM. Three independent mouse infection experiments were performed with similar trends; representative data are shown. One-way ANOVA with Dunnett’s multiple comparison test was used for statistical analysis, with mock samples used as a reference. *, *p* < 0.05, ** *p* < 0.01, *** *p* < 0.001, **** *p* < 0.0001.
(TIF)

S4 Fig Expression of CLRs infected neutrophils. Bone marrow-derived neutrophils of C57BL/6J mice were exposed to live bacteria (10 MOI). mRNA levels of select CLRs were analyzed via qRT-PCR. Data are presented relative to GAPDH values and shown as mean ± SEM. Unpaired t-test was used for statistical analysis, with Mock samples used as a reference. *, *p* < 0.05, ** *p* < 0.01.
(TIF)

S5 Fig Proinflammatory signatures and bacterial loads in WT and Mincle^-/-^ MΦ following infection. Bone marrow-derived WT or Mincle^-/-^ MΦ were exposed to live bacteria (MOI 5, or 10). qRT-PCR analyses of indicated genes at (A) 4 hr and (B) 24 hr post-infection are presented with data presented relative to GAPDH and shown as mean ± SEM. One-way ANOVA with Dunnett’s multiple comparison test was performed for treatment groups within the WT or Mincle^-/-^ MΦ background, respectively. Unpaired t-test was utilized for comparison between infected WT and Mincle^-/-^ MΦs. (C) Bacterial growth in infected MΦs (MOI 2 or 5) was analyzed at 4, 24, and 48 hr. Bacterial loads were determined by qPCR. Data are presented as the copy number of *O*. *tsutsugamushi* 47-kDa gene copy per ng of DNA. Unpaired t-test was used for statistical analysis. *, *p* < 0.05; **, *p* < 0.01; ***, *p* < 0.001; ****, *p* < 0.0001.
(TIF)

S6 Fig Late Effects of TNFα on Mincle transcription and exacerbation of Mincle-dependent inflammatory responses in MΦ exposed to *O*. *tsutsugamushi*. Bone marrow-derived WT or Mincle^-/-^ MΦ were primed with TNFα (25 ng/ml for 30 min) prior to infection with live bacteria (5 MOI). qRT-PCR analyses of indicated genes (relative to GAPDH) at 24 hr post-infection are presented; data are shown as mean ± SEM. One-way ANOVA with Dunnett’s multiple comparison test was performed for treatment groups within the WT or Mincle^-/-^ MΦ backgrounds, respectively. Unpaired t-test was utilized for comparison between infected WT and Mincle^-/-^ MΦs. *, *p* < 0.05; **, *p* < 0.01; ***, *p* < 0.001; ****, *p* < 0.0001.
(TIF)
