# Supplementary material for: Orientia tsutsugamushi selectively stimulates the C-type lectin receptor Mincle and type 1-skewed proinflammatory immune responses
Source: PLoS Pathog. 2021 Jul 28;17(7):e1009782. doi: 10.1371/journal.ppat.1009782 (PMC8351992; doi:10.1371/journal.ppat.1009782)
Supplement: S1 Table — (DOCX) [file ppat.1009782.s001.docx]

| Gene Target | Forward 5’-3’ | Reverse 5’-3 prime |
| --- | --- | --- |
| *Ahr* | AGCCGGTGCAGAAAACAGTAA | AGGCGGTCTAACTCTGTGTTC |
| *Ccl2* | GTGCTGACCCCAAGAAGGAA | GTGCTGAAGACCTTAGGGCA |
| *Ccl4* | TTCCTGCTGTTTCTCTTACACCT | CTGTCTGCCTCTTTTGGTCAG |
| *Ccl5* | GCTGCTTTGCCTACCTCTCC | TCGAGTGACAAACACGACTGC |
| *Ccl7* | GCTGCTTTCAGCATCCAAGTG | CCAGGGACACCGACTACTG |
| *Ccrl1* | AGCCAGTACGAAGTGATCTGC | CTGCGAGCCCAGTGACAAA |
| *Cxcl1* | CTGGGATTCACCTCAAGAACATC | CAGGGTCAAGGCAAGCCTC |
| *Cxcl9* | GGAGTTCGAGGAACCCTAGTG | GGGATTTGTAGTGGATCGTGC |
| *Cxcl10* | CCAAGTGCTGCCGTCATTTTC | GGCTCGCAGGGATGATTTCAA |
| *Cxcl12* | TGCATCAGTGACGGTAAACCA | CACAGTTTGGAGTGTTGAGGAT |
| *Clec4b1* | ACCAGTTTAGTATGGACAAGCCA | GTGGGAACCAAGTAGCAGTGG |
| *Clec4d* (MCL) | ACCCGACATCCCCAACTGAT | CTCTCGTCCAGCGTAAAAAGT |
| *Clec4e* (Mincle) | AGTGCTCTCCTGGACGATAG | CCTGATGCCTCACTGTAGCAG |
| *Clec5a* (MDL-1) | TCGGGGCTTATCGTAGTAGTG | TGTAGGCATGGTACTTTCGTCAT |
| *Clec6a* (Dectin1) | AAGCGGAGCAGAATTTCATCA | CCATTTGCCATTACCTTGTGGA |
| *Clec7a* (Dectin2) | GACTTCAGCACTCAAGACATCC | TTGTGTCGCCAAAATGCTAGG |
| *Clec9a* | GAAGTGCCAATCCCCTAGCAA | GCATCTGTGCCTGAATGGAGA |
| *Clec12a* | AGAAGTCTGACAAATGTGGGGG | CCAATGAACAGCAGAAGGCATA |
| *Clec13d* (Mrc-1) | CTCTGTTCAGCTATTGGACGC | CGGAATTTCTGGGATTCAGCTTC |
| *Fcgr1* | AGGTTCCTCAATGCCAAGTGA | GCGACCTCCGAATCTGAAGA |
| *Fcgr2b* | AGGGCCTCCATCTGGACTG | GTGGTTCTGGTAATCATGCTCTG |
| *Fcgr3* | CAGAATGCACACTCTGGAAGC | GGGTCCCTTCGCACATCAG |
| *Fcgr4* | ATGTGGCAGCTACTACTACCA | ACCCACTTGGGGTCTAGGTTC |
| *Fcer1g* | ATCTCAGCCGTGATCTTGTTCT | ACCATACAAAAACAGGACAGCAT |
| *Il27* | CTGTTGCTGCTACCCTTGCTT | CACTCCTGGCAATCGAGATTC |
| *Tnf* | CCCTCACACTCAGATCATCTTCT | GCTACGACGTGGGCTACAG |
| *Nos2* | GTTCTCAGCCCAACAATACAAGA | GTGGACGGGTCGATGTCAC |
| GAPDH | TGGAAAGCTGTGGCGTGAT | TGCTTCACCACCTTCTTGAT |
| *Orientia tsutsugamushi* 47-kDa | AACTGATTTTATTCAAACTAATGCTGCT | TATGCCTGAGTAAGATACTGTAATGGA |
