# Supplementary material for: Orientia tsutsugamushi selectively stimulates the C-type lectin receptor Mincle and type 1-skewed proinflammatory immune responses
Source: PLoS Pathog. 2021 Jul 28;17(7):e1009782. doi: 10.1371/journal.ppat.1009782 (PMC8351992; doi:10.1371/journal.ppat.1009782)
Supplement: S2 Table — (DOCX) [file ppat.1009782.s002.docx]

| **Supplemental Table 2. Complete list of differentially expressed genes in lung tissues (D2 vs. mock)**  *, unadjusted test statistic; **, adjusted test statistic via the Benjamini-Yekutieli procedure | | | | | |
| --- | --- | --- | --- | --- | --- |
| **Gene** | **Log2 fold change** | **std error (log2)** | **P-value*** | **BY.p.value**** | **probe.ID** |
| Cfd | 4.66 | 1.92 | 0.0728 | 1 | NM_013459.1:526 |
| H2-Q10 | 2.43 | 0.875 | 0.0497 | 1 | NM_010391.4:890 |
| Ptger3 | 2.36 | 0.843 | 0.0489 | 1 | NM_011196.2:1541 |
| Hamp | 2.32 | 1.12 | 0.108 | 1 | NM_032541.1:202 |
| Mef2a | 1.84 | 0.548 | 0.0283 | 1 | XM_976032.1:174 |
| Crp | 1.52 | 0.847 | 0.147 | 1 | NM_007768.4:163 |
| Ccr8 | 1.44 | 1.73 | 0.45 | 1 | NM_007720.2:426 |
| Klra1 | 1.4 | 0.789 | 0.15 | 1 | NM_016659.3:105 |
| Mafg | 1.15 | 0.239 | 0.00868 | 1 | XM_001002362.1:516 |
| Ccl11 | 1.01 | 0.31 | 0.0314 | 1 | NM_011330.3:430 |
| Defb1 | 1 | 0.878 | 0.318 | 1 | NM_007843.3:157 |
| Itln1 | 0.967 | 0.565 | 0.162 | 1 | NM_010584.3:853 |
| Cd163 | 0.953 | 0.383 | 0.0673 | 1 | NM_053094.2:3225 |
| Retnla | 0.953 | 0.531 | 0.147 | 1 | NM_020509.3:164 |
| Irf3 | 0.906 | 0.26 | 0.0251 | 1 | NM_016849.3:1527 |
| Ccl24 | 0.897 | 0.509 | 0.153 | 1 | NM_019577.4:335 |
| Klrc3 | 0.829 | 0.626 | 0.256 | 1 | NM_021378.1:242 |
| Tnfrsf4 | 0.823 | 0.134 | 0.0036 | 1 | NM_011659.2:320 |
| Ifna2 | 0.823 | 0.316 | 0.0599 | 1 | NM_010503.2:89 |
| Ltf | 0.816 | 0.688 | 0.301 | 1 | NM_008522.3:2545 |
| Pik3c2g | 0.778 | 0.512 | 0.203 | 1 | NM_011084.2:55 |
| Ikbke | 0.753 | 0.0811 | 0.000747 | 1 | NM_019777.3:2495 |
| Pdcd1lg2 | 0.747 | 0.215 | 0.0256 | 1 | NM_021396.2:1870 |
| Elk1 | 0.71 | 0.271 | 0.0587 | 1 | NM_007922.4:3070 |
| Il11ra1 | 0.709 | 0.219 | 0.0317 | 1 | NM_010549.3:22 |
| Frmpd4 | 0.707 | 0.464 | 0.203 | 1 | NM_001033330.2:4690 |
| Nox3 | 0.698 | 0.538 | 0.265 | 1 | NM_198958.2:1240 |
| Hras1 | 0.674 | 0.38 | 0.151 | 1 | NM_008284.2:1890 |
| Pparg | 0.636 | 0.209 | 0.0385 | 1 | NM_011146.1:1060 |
| Fxyd2 | 0.636 | 0.366 | 0.157 | 1 | NM_052823.2:248 |
| Mx1 | 0.633 | 0.239 | 0.0572 | 1 | NM_010846.1:2485 |
| Il17rb | 0.61 | 0.23 | 0.0566 | 1 | NM_019583.3:285 |
| Cfi | 0.61 | 0.554 | 0.332 | 1 | NM_007686.2:421 |
| H2-Ea-ps | 0.595 | 0.492 | 0.293 | 1 | NM_010381.2:735 |
| Masp1 | 0.561 | 0.41 | 0.243 | 1 | NM_008555.2:210 |
| Nfkbiz | 0.551 | 0.161 | 0.0266 | 1 | NM_030612.1:1305 |
| Ppp1r12b | 0.506 | 0.244 | 0.107 | 1 | NM_001081307.1:2560 |
| Fkbp5 | 0.481 | 0.443 | 0.338 | 1 | NM_010220.3:2125 |
| Cd209g | 0.461 | 0.29 | 0.187 | 1 | NM_027343.3:644 |
| Il22 | 0.45 | 0.404 | 0.328 | 1 | NM_016971.1:477 |
| Il12rb1 | 0.445 | 0.12 | 0.0206 | 1 | NM_008353.2:1757 |
| Cd46 | 0.443 | 0.307 | 0.222 | 1 | NM_010778.3:115 |
| Plcb1 | 0.441 | 0.168 | 0.0587 | 1 | NM_019677.1:495 |
| Cxcl5 | 0.436 | 0.519 | 0.448 | 1 | NM_009141.2:565 |
| Bcl2 | 0.432 | 0.227 | 0.13 | 1 | NM_009741.3:1844 |
| Map3k9 | 0.422 | 0.153 | 0.0515 | 1 | NM_177395.4:2695 |
| Il10 | 0.411 | 0.667 | 0.571 | 1 | NM_010548.1:985 |
| Il2.1 | 0.407 | 0.19 | 0.0988 | 1 | NM_008366.2:485 |
| Pdcd1 | 0.403 | 0.318 | 0.275 | 1 | NM_008798.1:1134 |
| Hspb2 | 0.393 | 0.249 | 0.189 | 1 | NM_024441.3:667 |
| Cd1d1 | 0.384 | 0.255 | 0.206 | 1 | NM_007639.3:1340 |
| Cd59b | 0.376 | 0.662 | 0.601 | 1 | NM_181858.1:310 |
| Mbl2 | 0.374 | 0.354 | 0.351 | 1 | NM_010776.1:525 |
| Csf3 | 0.369 | 0.426 | 0.435 | 1 | NM_009971.1:830 |
| Twist2 | 0.368 | 0.36 | 0.365 | 1 | NM_007855.2:1102 |
| Fas | 0.356 | 0.188 | 0.131 | 1 | NM_007987.2:95 |
| Mx2 | 0.356 | 0.305 | 0.308 | 1 | NM_013606.1:2095 |
| Ifi27l2a | 0.352 | 0.328 | 0.343 | 1 | NM_029803.1:270 |
| Jak3 | 0.35 | 0.178 | 0.12 | 1 | NM_010589.5:145 |
| Cul9 | 0.34 | 0.229 | 0.212 | 1 | NM_001081335.2:2552 |
| Mapk1 | 0.332 | 0.175 | 0.131 | 1 | NM_001038663.1:1490 |
| Ctla4 | 0.326 | 0.328 | 0.377 | 1 | NM_009843.3:1475 |
| Ikbkap | 0.318 | 0.187 | 0.164 | 1 | NM_026079.3:38 |
| Tslp | 0.307 | 0.393 | 0.478 | 1 | NM_021367.1:638 |
| Ncam1 | 0.298 | 0.369 | 0.465 | 1 | NM_001113204.1:740 |
| Selplg | 0.294 | 0.56 | 0.628 | 1 | NM_009151.3:2210 |
| Il3 | 0.284 | 0.229 | 0.282 | 1 | NM_010556.4:155 |
| Btla | 0.269 | 0.199 | 0.247 | 1 | NM_177584.3:2050 |
| Tlr6 | 0.253 | 0.137 | 0.138 | 1 | NM_011604.3:475 |
| Creb1 | 0.248 | 0.435 | 0.599 | 1 | NM_133828.2:7980 |
| Icosl | 0.245 | 0.178 | 0.241 | 1 | NM_015790.3:349 |
| Bcl2l1 | 0.244 | 0.206 | 0.302 | 1 | NM_009743.4:200 |
| Traf1 | 0.243 | 0.177 | 0.242 | 1 | NM_009421.3:1420 |
| Runx3 | 0.241 | 0.245 | 0.381 | 1 | NM_019732.2:100 |
| Birc2 | 0.238 | 0.13 | 0.141 | 1 | NM_007465.2:1230 |
| Il1rl1 | 0.236 | 0.246 | 0.391 | 1 | NM_001025602.2:815 |
| Ifitm1 | 0.229 | 0.147 | 0.194 | 1 | NM_001112715.1:412 |
| Il15ra | 0.229 | 0.193 | 0.303 | 1 | NM_008358.2:800 |
| Adal | 0.227 | 0.179 | 0.275 | 1 | NM_029475.1:1202 |
| Gpr44 | 0.225 | 0.261 | 0.438 | 1 | NM_009962.2:270 |
| Ptgfr | 0.224 | 0.343 | 0.549 | 1 | NM_008966.3:1202 |
| C4bp | 0.217 | 0.338 | 0.555 | 1 | NM_007576.3:580 |
| Ptpn22 | 0.2 | 0.128 | 0.193 | 1 | NM_008979.1:1203 |
| Stat2 | 0.198 | 0.268 | 0.5 | 1 | NM_019963.1:1955 |
| Fn1 | 0.194 | 0.385 | 0.641 | 1 | NM_010233.1:2627 |
| Atg16l1 | 0.188 | 0.139 | 0.247 | 1 | NM_029846.3:1250 |
| Card9 | 0.187 | 0.143 | 0.26 | 1 | NM_001037747.1:1227 |
| Cxcl13 | 0.184 | 0.308 | 0.582 | 1 | NM_018866.2:551 |
| Psmb11 | 0.183 | 0.412 | 0.679 | 1 | NM_175204.4:3953 |
| Batf3 | 0.181 | 0.281 | 0.555 | 1 | NM_030060.2:345 |
| Il15 | 0.178 | 0.25 | 0.515 | 1 | NM_008357.1:205 |
| Cd53 | 0.171 | 0.255 | 0.54 | 1 | NM_007651.3:2300 |
| Chuk | 0.166 | 0.138 | 0.296 | 1 | NM_001162410.1:2240 |
| Klra5 | 0.164 | 0.415 | 0.713 | 1 | NM_008463.2:174 |
| Foxp3 | 0.163 | 0.359 | 0.672 | 1 | NM_054039.1:150 |
| Nod1 | 0.16 | 0.175 | 0.411 | 1 | NM_172729.2:1446 |
| Cd19 | 0.16 | 0.296 | 0.617 | 1 | NM_009844.2:1697 |
| Icam5 | 0.157 | 0.237 | 0.545 | 1 | NM_008319.2:1867 |
| Notch2 | 0.155 | 0.217 | 0.515 | 1 | NM_010928.1:5110 |
| Klra6 | 0.152 | 0.319 | 0.658 | 1 | NM_008464.2:880 |
| Fcamr | 0.148 | 0.28 | 0.624 | 1 | NM_001170632.1:542 |
| Il23r | 0.147 | 0.189 | 0.479 | 1 | NM_144548.1:690 |
| Il22ra2 | 0.143 | 0.287 | 0.644 | 1 | NM_178258.5:20 |
| Folr4 | 0.143 | 0.323 | 0.682 | 1 | NM_022888.2:245 |
| Tyk2 | 0.139 | 0.184 | 0.491 | 1 | NM_018793.2:3465 |
| Il27 | 0.138 | 0.407 | 0.752 | 1 | NM_145636.1:175 |
| Lilra6 | 0.137 | 0.3 | 0.672 | 1 | NM_011090.2:424 |
| Casp1 | 0.134 | 0.197 | 0.532 | 1 | NM_009807.2:259 |
| Mapk8 | 0.132 | 0.155 | 0.442 | 1 | NM_016700.3:970 |
| Atm | 0.132 | 0.224 | 0.587 | 1 | NM_007499.1:7840 |
| Ikbkb | 0.131 | 0.14 | 0.401 | 1 | NM_010546.2:4280 |
| Raf1 | 0.128 | 0.111 | 0.313 | 1 | NM_029780.3:550 |
| Cd96 | 0.115 | 0.146 | 0.475 | 1 | NM_032465.2:34 |
| Nfatc2 | 0.115 | 0.223 | 0.633 | 1 | NM_001037177.1:1559 |
| Igf2r | 0.115 | 0.239 | 0.656 | 1 | NM_010515.1:2585 |
| Lilrb4 | 0.106 | 0.06 | 0.152 | 1 | NM_013532.2:1262 |
| Pdgfrb | 0.104 | 0.242 | 0.69 | 1 | NM_008809.1:1185 |
| Gm10499 | 0.104 | 0.336 | 0.772 | 1 | XM_003086920.1:898 |
| Traf4 | 0.103 | 0.198 | 0.631 | 1 | NM_009423.4:2210 |
| Tnfrsf17 | 0.1 | 0.404 | 0.816 | 1 | NM_011608.1:140 |
| Cxcl9 | 0.0984 | 0.318 | 0.773 | 1 | NM_008599.2:40 |
| Alox15 | 0.0971 | 0.423 | 0.83 | 1 | NM_009660.3:1419 |
| Btnl2 | 0.096 | 0.516 | 0.861 | 1 | NM_079835.2:300 |
| Il7 | 0.094 | 0.26 | 0.736 | 1 | NM_008371.2:1055 |
| Ccl19 | 0.0914 | 0.21 | 0.685 | 1 | NM_011888.2:465 |
| Alox5 | 0.0862 | 0.239 | 0.737 | 1 | NM_009662.2:1206 |
| Stat5a | 0.0815 | 0.17 | 0.656 | 1 | NM_011488.2:1545 |
| Mapk11 | 0.0717 | 0.242 | 0.782 | 1 | NM_011161.5:2107 |
| Nfkbia | 0.0688 | 0.205 | 0.754 | 1 | NM_010907.2:646 |
| Cd109 | 0.0654 | 0.396 | 0.877 | 1 | NM_153098.3:2720 |
| Tlr1 | 0.0627 | 0.225 | 0.794 | 1 | NM_030682.1:805 |
| Myl2 | 0.0593 | 0.518 | 0.914 | 1 | NM_010861.3:205 |
| Lef1 | 0.0592 | 0.183 | 0.762 | 1 | NM_010703.3:1337 |
| Tnfaip6 | 0.059 | 0.215 | 0.797 | 1 | NM_009398.2:620 |
| Hdac4 | 0.0563 | 0.187 | 0.778 | 1 | NM_207225.1:2800 |
| Prkca | 0.0502 | 0.254 | 0.853 | 1 | NM_011101.3:6965 |
| Cdkn1a | 0.0475 | 0.22 | 0.839 | 1 | NM_007669.4:1670 |
| Defb14 | 0.0474 | 0.415 | 0.915 | 1 | NM_183026.2:128 |
| Fcgr2b | 0.0445 | 0.093 | 0.657 | 1 | NM_001077189.1:1225 |
| Fcer1a | 0.0384 | 0.378 | 0.924 | 1 | NM_010184.1:114 |
| Irak3 | 0.0361 | 0.0356 | 0.368 | 1 | NM_028679.3:921 |
| Il6ra | 0.0342 | 0.216 | 0.882 | 1 | NM_010559.2:2825 |
| Map3k5 | 0.0338 | 0.164 | 0.847 | 1 | NM_008580.4:640 |
| Prim1 | 0.0337 | 0.174 | 0.856 | 1 | NM_008921.2:1166 |
| Irak1 | 0.0317 | 0.108 | 0.785 | 1 | NM_008363.2:951 |
| Notch1 | 0.0269 | 0.301 | 0.933 | 1 | NM_008714.2:1425 |
| H2-Ob | 0.0259 | 0.32 | 0.939 | 1 | NM_010389.3:1205 |
| C3ar1 | 0.0238 | 0.203 | 0.913 | 1 | NM_009779.2:555 |
| Tbk1 | 0.0204 | 0.19 | 0.92 | 1 | NM_019786.4:440 |
| C1qb | 0.0193 | 0.103 | 0.86 | 1 | NM_009777.2:865 |
| Tnfsf8 | 0.0188 | 0.328 | 0.957 | 1 | NM_009403.2:125 |
| Rae1 | 0.0161 | 0.0913 | 0.869 | 1 | NM_175112.5:1485 |
| Prkcd | 0.0125 | 0.0738 | 0.874 | 1 | NM_011103.2:1265 |
| Abcb10 | 0.0102 | 0.15 | 0.949 | 1 | NM_019552.2:1090 |
| Limk1 | 0.0102 | 0.169 | 0.955 | 1 | NM_010717.2:1583 |
| Cfp | 0.0102 | 0.172 | 0.956 | 1 | NM_008823.3:1107 |
| C3 | 0.00993 | 0.161 | 0.954 | 1 | NM_009778.2:285 |
| Irf4 | 0.00959 | 0.199 | 0.964 | 1 | NM_013674.1:1878 |
| Cd40 | 0.00708 | 0.176 | 0.97 | 1 | NM_011611.2:1425 |
| Cd244 | 0.00614 | 0.18 | 0.974 | 1 | NM_018729.2:262 |
| Maf | 0.00609 | 0.214 | 0.979 | 1 | NM_001025577.2:43 |
| Map3k7 | 0.00419 | 0.142 | 0.978 | 1 | NM_172688.2:2405 |
| Bcl6 | 0.00401 | 0.111 | 0.973 | 1 | NM_009744.3:185 |
| Smad3 | -0.00175 | 0.178 | 0.993 | 1 | NM_016769.3:1845 |
| Hspb1 | -0.00238 | 0.124 | 0.986 | 1 | NM_013560.2:630 |
| Pigr | -0.00945 | 0.447 | 0.984 | 1 | NM_011082.3:585 |
| Tnfrsf14 | -0.0163 | 0.162 | 0.925 | 1 | NM_178931.2:625 |
| Mr1 | -0.0181 | 0.239 | 0.943 | 1 | NM_008209.4:1360 |
| Il21 | -0.0198 | 0.49 | 0.97 | 1 | NM_021782.2:1762 |
| C1ra | -0.0232 | 0.187 | 0.907 | 1 | NM_023143.3:64 |
| Ccl8 | -0.0236 | 0.458 | 0.961 | 1 | NM_021443.2:150 |
| Mef2b | -0.025 | 0.477 | 0.961 | 1 | NM_001045484.1:1091 |
| Mrc1 | -0.0254 | 0.162 | 0.883 | 1 | NM_008625.1:3992 |
| C6 | -0.0266 | 0.275 | 0.928 | 1 | NM_016704.2:1135 |
| Il20 | -0.0284 | 0.705 | 0.97 | 1 | NM_021380.1:375 |
| Sigirr | -0.0292 | 0.191 | 0.886 | 1 | NM_023059.3:800 |
| Tlr5 | -0.034 | 0.234 | 0.891 | 1 | NM_016928.2:560 |
| Tnfrsf11a | -0.0381 | 0.151 | 0.813 | 1 | NM_009399.3:3870 |
| Trp53 | -0.0382 | 0.0728 | 0.627 | 1 | NM_011640.1:1835 |
| Ptafr | -0.0389 | 0.153 | 0.812 | 1 | NM_001081211.1:1685 |
| Mknk1 | -0.0396 | 0.191 | 0.846 | 1 | NM_021461.4:635 |
| Mif | -0.0415 | 0.169 | 0.818 | 1 | NM_010798.2:373 |
| Ebi3 | -0.0432 | 0.151 | 0.79 | 1 | NM_015766.2:1015 |
| Ccl9 | -0.0435 | 0.251 | 0.871 | 1 | NM_011338.2:1125 |
| Pla2g4a | -0.0471 | 0.134 | 0.742 | 1 | NM_008869.2:1525 |
| Cmklr1 | -0.0492 | 0.165 | 0.78 | 1 | NM_008153.3:445 |
| Il15.1 | -0.0499 | 0.289 | 0.871 | 1 | NM_008357.2:854 |
| Lta | -0.0506 | 0.353 | 0.893 | 1 | NM_010735.1:1115 |
| Kir3dl2 | -0.052 | 0.363 | 0.893 | 1 | NM_177748.2:1519 |
| Ncf4 | -0.0537 | 0.178 | 0.778 | 1 | NM_008677.2:741 |
| Tbxa2r | -0.0541 | 0.112 | 0.654 | 1 | NM_001277265.1:477 |
| Mef2d | -0.0552 | 0.157 | 0.743 | 1 | NM_133665.3:1890 |
| Psmc2 | -0.0578 | 0.0558 | 0.359 | 1 | NM_011188.3:654 |
| Nfatc1 | -0.0579 | 0.189 | 0.775 | 1 | NM_016791.4:1570 |
| Ikzf1 | -0.0596 | 0.16 | 0.728 | 1 | NM_001025597.1:4420 |
| Cish | -0.0596 | 0.169 | 0.741 | 1 | NM_009895.3:1541 |
| Il16 | -0.0597 | 0.152 | 0.714 | 1 | NM_010551.3:3095 |
| Map4k2 | -0.0597 | 0.169 | 0.742 | 1 | NM_009006.2:666 |
| Ddit3 | -0.06 | 0.167 | 0.738 | 1 | NM_007837.3:255 |
| Mbp | -0.0601 | 0.183 | 0.759 | 1 | NM_010777.3:761 |
| Pml | -0.0602 | 0.144 | 0.697 | 1 | NM_008884.2:2765 |
| Rps6ka5 | -0.0606 | 0.141 | 0.69 | 1 | NM_153587.2:2755 |
| Irak2 | -0.0617 | 0.0809 | 0.488 | 1 | NM_001113553.1:485 |
| Cradd | -0.0622 | 0.112 | 0.608 | 1 | NM_009950.2:1470 |
| Hsh2d | -0.0632 | 0.183 | 0.747 | 1 | NM_197944.1:1023 |
| Mapk14 | -0.0637 | 0.12 | 0.625 | 1 | NM_011951.2:1420 |
| Jak2 | -0.0639 | 0.145 | 0.682 | 1 | NM_001048177.1:730 |
| Marco | -0.0661 | 0.178 | 0.729 | 1 | NM_010766.2:350 |
| C1qa | -0.0662 | 0.145 | 0.671 | 1 | NM_007572.2:566 |
| Ripk2 | -0.0666 | 0.179 | 0.728 | 1 | NM_138952.3:830 |
| Bax | -0.0677 | 0.164 | 0.7 | 1 | NM_007527.3:735 |
| Irak4 | -0.0678 | 0.131 | 0.631 | 1 | NM_029926.5:250 |
| Gpi1 | -0.0678 | 0.225 | 0.778 | 1 | NM_008155.3:2675 |
| Ripk1 | -0.0685 | 0.149 | 0.669 | 1 | NM_009068.3:3185 |
| Atf2 | -0.069 | 0.136 | 0.639 | 1 | NM_001025093.1:3995 |
| Abl1 | -0.0691 | 0.182 | 0.723 | 1 | NM_009594.3:4216 |
| Bid | -0.0696 | 0.0535 | 0.263 | 1 | NM_007544.3:1307 |
| Ptk2 | -0.0706 | 0.208 | 0.751 | 1 | NM_007982.2:1060 |
| Rock2 | -0.0736 | 0.17 | 0.687 | 1 | NM_009072.2:4680 |
| Max | -0.074 | 0.105 | 0.519 | 1 | NM_008558.1:120 |
| Traf5 | -0.0744 | 0.168 | 0.68 | 1 | NM_011633.1:1940 |
| Ceacam1 | -0.0749 | 0.184 | 0.706 | 1 | NM_001039185.1:294 |
| Klra21 | -0.0767 | 0.649 | 0.912 | 1 | NM_053151.1:41 |
| Zap70 | -0.0768 | 0.0779 | 0.38 | 1 | NM_009539.2:1030 |
| Ccrl2 | -0.0777 | 0.183 | 0.694 | 1 | NM_017466.4:655 |
| Prdm1 | -0.0779 | 0.197 | 0.713 | 1 | NM_007548.3:1440 |
| Smad5 | -0.0782 | 0.163 | 0.657 | 1 | NM_008541.2:2630 |
| Hc | -0.0791 | 0.192 | 0.702 | 1 | NM_010406.1:1065 |
| Il1rl2 | -0.0809 | 0.251 | 0.764 | 1 | NM_133193.3:860 |
| Tlr3 | -0.0811 | 0.222 | 0.733 | 1 | NM_126166.2:1165 |
| Tnfaip3 | -0.0818 | 0.148 | 0.609 | 1 | NM_009397.2:232 |
| Map2k4 | -0.0837 | 0.141 | 0.584 | 1 | NM_009157.4:1335 |
| Traf3 | -0.0839 | 0.174 | 0.655 | 1 | NM_001048206.1:6385 |
| Trem2 | -0.085 | 0.225 | 0.725 | 1 | NM_031254.2:646 |
| Kng1 | -0.0853 | 0.471 | 0.865 | 1 | NM_023125.3:1545 |
| Rag2 | -0.0857 | 0.346 | 0.817 | 1 | NM_009020.3:1318 |
| C1qbp | -0.0874 | 0.0974 | 0.42 | 1 | NM_007573.2:630 |
| Map4k1 | -0.0876 | 0.132 | 0.544 | 1 | NM_008279.2:1758 |
| Ptpn2 | -0.088 | 0.132 | 0.543 | 1 | NM_001127177.1:160 |
| Npc1 | -0.0883 | 0.185 | 0.658 | 1 | NM_008720.2:2645 |
| Ccbp2 | -0.0893 | 0.286 | 0.77 | 1 | NM_021609.3:1765 |
| Nox4 | -0.0903 | 0.199 | 0.674 | 1 | NM_015760.4:1535 |
| Psmb5 | -0.0916 | 0.142 | 0.554 | 1 | NM_011186.1:334 |
| Il1r1 | -0.092 | 0.132 | 0.526 | 1 | NM_001123382.1:820 |
| Zbtb7b | -0.0941 | 0.0972 | 0.388 | 1 | NM_009565.4:2178 |
| Cd6 | -0.0941 | 0.177 | 0.623 | 1 | NM_001037801.2:1315 |
| Gnas | -0.0964 | 0.0697 | 0.239 | 1 | NM_010309.3:2592 |
| Map2k6 | -0.0974 | 0.188 | 0.632 | 1 | NM_011943.2:320 |
| Xbp1 | -0.098 | 0.164 | 0.582 | 1 | NM_013842.2:825 |
| Blnk | -0.098 | 0.191 | 0.635 | 1 | NM_008528.4:1546 |
| Irgm1 | -0.098 | 0.258 | 0.723 | 1 | NM_008326.1:27 |
| Ccr5 | -0.0989 | 0.147 | 0.537 | 1 | NM_009917.5:1340 |
| Tlr8 | -0.0992 | 0.17 | 0.59 | 1 | NM_133212.2:110 |
| Tradd | -0.0997 | 0.159 | 0.564 | 1 | NM_001033161.2:562 |
| Ifi204 | -0.101 | 0.165 | 0.573 | 1 | NM_008329.2:1296 |
| Ltb4r1 | -0.103 | 0.227 | 0.674 | 1 | NM_008519.2:125 |
| Ptger2 | -0.105 | 0.174 | 0.58 | 1 | NM_008964.4:2000 |
| Cebpb | -0.107 | 0.156 | 0.532 | 1 | NM_009883.3:1147 |
| Psmd7 | -0.109 | 0.032 | 0.0271 | 1 | NM_010817.2:1424 |
| Il10ra | -0.109 | 0.0759 | 0.224 | 1 | NM_008348.2:75 |
| Lilrb3 | -0.109 | 0.0882 | 0.283 | 1 | NM_011095.2:2040 |
| Myd88 | -0.109 | 0.123 | 0.424 | 1 | NM_010851.2:1595 |
| Phlpp2 | -0.109 | 0.19 | 0.594 | 1 | NM_001122594.2:7105 |
| Cr2 | -0.11 | 0.42 | 0.806 | 1 | NM_007758.2:1650 |
| Il27ra | -0.111 | 0.2 | 0.607 | 1 | NM_016671.3:2320 |
| Ccl17 | -0.114 | 0.347 | 0.759 | 1 | NM_011332.2:247 |
| Tlr4 | -0.116 | 0.135 | 0.439 | 1 | NM_021297.2:2510 |
| C1s | -0.116 | 0.164 | 0.52 | 1 | NM_144938.2:2490 |
| Map3k1 | -0.116 | 0.206 | 0.604 | 1 | NM_011945.2:1640 |
| Pax5 | -0.118 | 0.31 | 0.722 | 1 | NM_008782.2:90 |
| Il21r | -0.118 | 0.33 | 0.739 | 1 | NM_021887.1:619 |
| Shc1 | -0.12 | 0.0545 | 0.0931 | 1 | NM_011368.4:235 |
| Oasl1 | -0.12 | 0.243 | 0.647 | 1 | NM_145209.2:1165 |
| Cxcr2 | -0.121 | 0.256 | 0.661 | 1 | NM_009909.3:440 |
| Stat6 | -0.122 | 0.12 | 0.365 | 1 | NM_009284.2:3465 |
| Cysltr1 | -0.122 | 0.192 | 0.558 | 1 | NM_021476.4:164 |
| Casp2 | -0.123 | 0.167 | 0.503 | 1 | NM_007610.1:2770 |
| Traf2 | -0.124 | 0.0912 | 0.246 | 1 | NM_009422.2:262 |
| Il7r | -0.125 | 0.179 | 0.524 | 1 | NM_008372.3:1020 |
| Stat5b | -0.126 | 0.16 | 0.475 | 1 | NM_011489.3:4855 |
| Clec4a4 | -0.127 | 0.494 | 0.81 | 1 | NM_001005860.2:722 |
| Ilf3 | -0.128 | 0.175 | 0.503 | 1 | NM_010561.2:1902 |
| Il12a | -0.129 | 0.268 | 0.656 | 1 | NM_008351.1:355 |
| Masp2 | -0.13 | 0.292 | 0.678 | 1 | NM_010767.3:363 |
| Smad7 | -0.131 | 0.191 | 0.529 | 1 | NM_001042660.1:3557 |
| Ikzf4 | -0.131 | 0.206 | 0.561 | 1 | NM_011772.2:2057 |
| C6.1 | -0.132 | 0.246 | 0.62 | 1 | NM_016704.2:170 |
| Cd81 | -0.133 | 0.11 | 0.292 | 1 | NM_133655.2:575 |
| Cd3eap | -0.135 | 0.15 | 0.419 | 1 | NM_145822.2:1858 |
| Ptger4 | -0.135 | 0.219 | 0.572 | 1 | NM_008965.1:315 |
| Psmb7 | -0.136 | 0.0934 | 0.218 | 1 | NM_011187.1:184 |
| Ptgs1 | -0.136 | 0.179 | 0.491 | 1 | NM_008969.3:1642 |
| Ikzf2 | -0.139 | 0.176 | 0.475 | 1 | NM_011770.4:7230 |
| Csf2 | -0.14 | 0.246 | 0.599 | 1 | NM_009969.4:452 |
| Mapk1.1 | -0.141 | 0.138 | 0.364 | 1 | NM_011949.3:1210 |
| Il4ra | -0.141 | 0.16 | 0.428 | 1 | NM_001008700.3:670 |
| Nfe2l2 | -0.142 | 0.148 | 0.391 | 1 | NM_010902.3:1665 |
| Clu | -0.143 | 0.22 | 0.551 | 1 | NM_013492.2:354 |
| Nfatc3 | -0.144 | 0.135 | 0.348 | 1 | NM_010901.2:2260 |
| Stat4 | -0.145 | 0.103 | 0.231 | 1 | NM_011487.4:1816 |
| Irf1 | -0.145 | 0.189 | 0.485 | 1 | NM_008390.1:365 |
| Cd24a | -0.145 | 0.215 | 0.538 | 1 | NM_009846.2:584 |
| Map2k1 | -0.147 | 0.0792 | 0.137 | 1 | NM_008927.3:1695 |
| Cd55 | -0.147 | 0.151 | 0.384 | 1 | NM_010016.2:1058 |
| Pla2g2e | -0.147 | 0.227 | 0.554 | 1 | NM_012044.2:224 |
| Stat1 | -0.148 | 0.243 | 0.577 | 1 | NM_009283.3:1590 |
| Ifngr1 | -0.151 | 0.177 | 0.441 | 1 | NM_010511.2:985 |
| Tnfsf15 | -0.152 | 0.121 | 0.28 | 1 | NM_177371.3:4695 |
| Jak1 | -0.154 | 0.185 | 0.453 | 1 | NM_146145.2:4080 |
| Keap1 | -0.156 | 0.0918 | 0.164 | 1 | NM_016679.4:4140 |
| Ikbkg | -0.157 | 0.161 | 0.384 | 1 | NM_178590.2:525 |
| Tmem173 | -0.158 | 0.091 | 0.158 | 1 | NM_028261.1:130 |
| Nfkb1 | -0.158 | 0.145 | 0.335 | 1 | NM_008689.2:2125 |
| Ptgir | -0.158 | 0.179 | 0.427 | 1 | NM_008967.3:1731 |
| Il1rap.1 | -0.158 | 0.247 | 0.557 | 1 | NM_134103.2:945 |
| Litaf | -0.159 | 0.138 | 0.314 | 1 | NM_019980.1:1100 |
| Hif1a | -0.161 | 0.149 | 0.339 | 1 | NM_010431.2:1294 |
| Serping1 | -0.162 | 0.192 | 0.444 | 1 | NM_009776.3:1480 |
| Rapgef2 | -0.162 | 0.2 | 0.462 | 1 | NM_001099624.2:5580 |
| Mapk3 | -0.164 | 0.166 | 0.381 | 1 | NM_011952.2:825 |
| Ccl22 | -0.164 | 0.241 | 0.533 | 1 | NM_009137.2:1096 |
| Irf5 | -0.165 | 0.0741 | 0.0897 | 1 | NM_012057.3:1826 |
| Tnfsf12 | -0.167 | 0.199 | 0.449 | 1 | NM_011614.3:1215 |
| Kit | -0.167 | 0.299 | 0.607 | 1 | NM_001122733.1:4275 |
| Ifih1 | -0.168 | 0.159 | 0.349 | 1 | NM_027835.2:1997 |
| Tirap | -0.169 | 0.133 | 0.272 | 1 | NM_001177847.1:200 |
| Ciita | -0.169 | 0.218 | 0.481 | 1 | NM_007575.2:3988 |
| Cfh | -0.169 | 0.235 | 0.512 | 1 | NM_009888.3:807 |
| Runx1 | -0.171 | 0.196 | 0.431 | 1 | NM_001111021.1:3055 |
| Rhoa | -0.172 | 0.106 | 0.182 | 1 | NM_016802.4:1885 |
| Il13ra1 | -0.174 | 0.173 | 0.372 | 1 | NM_133990.4:845 |
| Ddx58 | -0.174 | 0.181 | 0.39 | 1 | NM_172689.3:1751 |
| Tcf4 | -0.174 | 0.198 | 0.429 | 1 | NM_013685.1:3045 |
| Ube2l3 | -0.176 | 0.0672 | 0.0587 | 1 | NM_009456.2:1817 |
| Cxcl11 | -0.177 | 0.471 | 0.726 | 1 | NM_019494.1:345 |
| Hfe | -0.178 | 0.0915 | 0.124 | 1 | NM_010424.4:1505 |
| Rorc | -0.179 | 0.21 | 0.442 | 1 | NM_011281.2:1687 |
| Casp3 | -0.182 | 0.121 | 0.207 | 1 | NM_009810.2:630 |
| Abcf1 | -0.182 | 0.21 | 0.435 | 1 | NM_013854.1:875 |
| Csf3r | -0.183 | 0.195 | 0.4 | 1 | NM_001252651.1:1294 |
| Irf7 | -0.187 | 0.23 | 0.461 | 1 | NM_016850.2:705 |
| Casp8 | -0.188 | 0.117 | 0.184 | 1 | NM_009812.2:1463 |
| Ly96 | -0.188 | 0.167 | 0.323 | 1 | NM_016923.1:368 |
| Bcap31 | -0.19 | 0.0702 | 0.0534 | 1 | NM_012060.4:65 |
| Oas2 | -0.192 | 0.344 | 0.606 | 1 | NM_145227.2:3438 |
| Fcgrt | -0.195 | 0.119 | 0.177 | 1 | NM_010189.3:750 |
| Ccr1 | -0.195 | 0.191 | 0.366 | 1 | NM_009912.4:1526 |
| Tnfrsf8 | -0.197 | 0.45 | 0.684 | 1 | NM_009401.2:1275 |
| Fyn | -0.198 | 0.172 | 0.313 | 1 | NM_008054.2:1030 |
| Cd79a | -0.198 | 0.31 | 0.559 | 1 | NM_007655.3:1175 |
| Ccr2 | -0.199 | 0.145 | 0.241 | 1 | NM_009915.2:2965 |
| Il17ra | -0.2 | 0.102 | 0.122 | 1 | NM_008359.1:312 |
| Tagap | -0.2 | 0.139 | 0.226 | 1 | NM_145968.2:350 |
| Nfil3 | -0.2 | 0.208 | 0.39 | 1 | NM_017373.3:1299 |
| Btk | -0.202 | 0.113 | 0.147 | 1 | NM_013482.2:2255 |
| Ski | -0.202 | 0.184 | 0.333 | 1 | NM_011385.2:1210 |
| C9 | -0.202 | 0.339 | 0.584 | 1 | NM_013485.1:226 |
| Grb2 | -0.204 | 0.0769 | 0.0567 | 1 | NM_008163.3:1783 |
| Ifit2 | -0.205 | 0.182 | 0.324 | 1 | NM_008332.2:230 |
| Cd86 | -0.206 | 0.147 | 0.234 | 1 | NM_019388.3:251 |
| Il6 | -0.207 | 0.0928 | 0.0899 | 1 | NM_031168.1:40 |
| Cxcr3 | -0.208 | 0.0966 | 0.0979 | 1 | NM_009910.2:605 |
| Stat3 | -0.208 | 0.195 | 0.347 | 1 | NM_213659.2:2130 |
| Ltbr | -0.21 | 0.128 | 0.176 | 1 | NM_010736.3:1962 |
| Gnb1 | -0.211 | 0.0947 | 0.0902 | 1 | NM_008142.3:311 |
| Flt1 | -0.211 | 0.218 | 0.388 | 1 | NM_010228.3:1550 |
| Arg1 | -0.211 | 0.333 | 0.561 | 1 | NM_007482.3:626 |
| Tgfbi | -0.213 | 0.178 | 0.297 | 1 | NM_009369.4:1295 |
| Rela | -0.214 | 0.107 | 0.116 | 1 | NM_009045.4:645 |
| Cd97 | -0.215 | 0.217 | 0.377 | 1 | NM_011925.1:1975 |
| Myc | -0.216 | 0.18 | 0.296 | 1 | NM_010849.4:630 |
| Oas1a | -0.216 | 0.203 | 0.347 | 1 | NM_145211.2:471 |
| H2-K1 | -0.219 | 0.0763 | 0.0453 | 1 | NM_001001892.2:1370 |
| Fcgr3 | -0.22 | 0.166 | 0.257 | 1 | NM_010188.5:1175 |
| Tgfb3 | -0.22 | 0.239 | 0.408 | 1 | NM_009368.2:2410 |
| Tigit | -0.22 | 0.416 | 0.624 | 1 | NM_001146325.1:730 |
| Il12b | -0.221 | 0.183 | 0.293 | 1 | NM_008352.1:1045 |
| Il2ra | -0.221 | 0.21 | 0.352 | 1 | NM_008367.2:325 |
| Il28a | -0.222 | 0.642 | 0.746 | 1 | NM_001024673.2:72 |
| Nfkb2 | -0.223 | 0.0603 | 0.0209 | 1 | NM_019408.2:1150 |
| Ifnar2 | -0.223 | 0.0807 | 0.0505 | 1 | NM_001110498.1:725 |
| Ifi35 | -0.224 | 0.137 | 0.178 | 1 | NM_027320.4:820 |
| Pdgfa | -0.224 | 0.156 | 0.225 | 1 | NM_008808.3:805 |
| Csf1r | -0.225 | 0.17 | 0.257 | 1 | NM_001037859.1:3655 |
| Rac1 | -0.226 | 0.124 | 0.142 | 1 | NM_009007.2:1045 |
| Psmb10 | -0.226 | 0.141 | 0.185 | 1 | NM_013640.3:401 |
| Gnaq | -0.226 | 0.188 | 0.296 | 1 | NM_008139.5:3080 |
| Cd34 | -0.227 | 0.195 | 0.31 | 1 | NM_001111059.1:560 |
| Ccl7 | -0.228 | 0.163 | 0.235 | 1 | NM_013654.2:215 |
| Tap1 | -0.229 | 0.21 | 0.336 | 1 | NM_001161730.1:856 |
| Tnfsf18 | -0.229 | 0.244 | 0.402 | 1 | NM_183391.3:1445 |
| Tgfbr2 | -0.232 | 0.159 | 0.218 | 1 | NM_009371.2:475 |
| Il1r2 | -0.232 | 0.512 | 0.674 | 1 | NM_010555.4:1077 |
| Cd99 | -0.234 | 0.191 | 0.289 | 1 | NM_025584.2:716 |
| Hif1a.1 | -0.234 | 0.206 | 0.32 | 1 | NM_010431.1:2335 |
| Cd8a | -0.234 | 0.231 | 0.369 | 1 | NM_001081110.2:355 |
| Mmp9 | -0.234 | 0.406 | 0.596 | 1 | NM_013599.2:1570 |
| Il18r1 | -0.235 | 0.176 | 0.254 | 1 | NM_001161842.1:620 |
| Crlf2 | -0.236 | 0.115 | 0.109 | 1 | NM_001164735.1:1225 |
| Il18 | -0.236 | 0.242 | 0.385 | 1 | NM_008360.1:100 |
| Tcf7 | -0.238 | 0.181 | 0.259 | 1 | NM_009331.3:1810 |
| Hlx | -0.241 | 0.195 | 0.283 | 1 | NM_008250.2:1825 |
| Ifnar1 | -0.241 | 0.198 | 0.29 | 1 | NM_010508.1:1195 |
| Il18.1 | -0.242 | 0.204 | 0.302 | 1 | NM_008360.1:270 |
| Irf8 | -0.243 | 0.153 | 0.188 | 1 | NM_008320.3:2274 |
| Il2 | -0.243 | 0.373 | 0.551 | 1 | NM_008366.3:314 |
| Il10rb | -0.244 | 0.0796 | 0.0374 | 1 | NM_008349.5:465 |
| B2m | -0.244 | 0.146 | 0.17 | 1 | NM_009735.3:177 |
| Ifnb1 | -0.245 | 0.146 | 0.169 | 1 | NM_010510.1:335 |
| Cd164 | -0.25 | 0.157 | 0.186 | 1 | NM_016898.2:688 |
| Ccr10 | -0.25 | 0.489 | 0.636 | 1 | NM_007721.4:1480 |
| Gngt1 | -0.252 | 0.354 | 0.516 | 1 | NM_010314.2:150 |
| Fadd | -0.254 | 0.228 | 0.328 | 1 | NM_010175.5:2641 |
| Prkcb | -0.254 | 0.283 | 0.421 | 1 | NM_008855.2:4220 |
| Cdc42 | -0.255 | 0.071 | 0.0228 | 1 | NM_009861.1:205 |
| H2-Eb1 | -0.255 | 0.147 | 0.159 | 1 | NM_010382.2:935 |
| Tlr9 | -0.256 | 0.2 | 0.27 | 1 | NM_031178.2:1801 |
| Tollip | -0.257 | 0.134 | 0.127 | 1 | NM_023764.3:260 |
| Mmp3 | -0.257 | 0.3 | 0.441 | 1 | NM_010809.1:1575 |
| Ccr6 | -0.257 | 0.35 | 0.504 | 1 | NM_001190333.1:660 |
| Slamf7 | -0.258 | 0.124 | 0.105 | 1 | NM_144539.5:750 |
| Phlpp1 | -0.258 | 0.204 | 0.273 | 1 | NM_133821.3:3246 |
| Src | -0.259 | 0.164 | 0.19 | 1 | NM_001025395.2:968 |
| Icam4 | -0.259 | 0.265 | 0.383 | 1 | NM_023892.2:540 |
| Mapkapk2 | -0.26 | 0.0652 | 0.0163 | 1 | NM_008551.1:1991 |
| Dpp4 | -0.26 | 0.141 | 0.139 | 1 | NM_001159543.1:1303 |
| Ccl2 | -0.263 | 0.144 | 0.142 | 1 | NM_011333.3:415 |
| Fasl | -0.263 | 0.15 | 0.154 | 1 | NM_010177.3:645 |
| H60a | -0.263 | 0.181 | 0.22 | 1 | NM_010400.2:2020 |
| Pdcd2 | -0.265 | 0.16 | 0.173 | 1 | NM_008799.2:837 |
| Psmb9 | -0.265 | 0.163 | 0.179 | 1 | NM_013585.2:540 |
| Cd8b1 | -0.265 | 0.25 | 0.35 | 1 | NM_009858.2:1075 |
| Entpd1 | -0.266 | 0.158 | 0.167 | 1 | NM_009848.3:170 |
| Ptpn6 | -0.267 | 0.0751 | 0.0236 | 1 | NM_013545.2:1691 |
| Fcgr4 | -0.267 | 0.198 | 0.249 | 1 | NM_144559.1:608 |
| Maff | -0.268 | 0.185 | 0.221 | 1 | NM_010755.3:743 |
| Xcr1 | -0.268 | 0.384 | 0.523 | 1 | NM_011798.4:350 |
| Tgfbr1 | -0.269 | 0.156 | 0.159 | 1 | NM_009370.2:4425 |
| Gfi1 | -0.269 | 0.276 | 0.385 | 1 | NM_010278.2:1875 |
| Tnfsf13b | -0.269 | 0.279 | 0.39 | 1 | NM_033622.1:225 |
| Cd14 | -0.27 | 0.192 | 0.232 | 1 | NM_009841.3:235 |
| Pdgfb | -0.273 | 0.176 | 0.196 | 1 | NM_011057.3:2325 |
| Hmgn1 | -0.275 | 0.12 | 0.084 | 1 | NM_008251.3:835 |
| Icos | -0.275 | 0.156 | 0.152 | 1 | NM_017480.1:142 |
| Tlr7 | -0.275 | 0.216 | 0.272 | 1 | NM_133211.3:3210 |
| Cd27 | -0.276 | 0.203 | 0.245 | 1 | NM_001042564.1:730 |
| Ets1 | -0.277 | 0.188 | 0.214 | 1 | NM_001038642.1:740 |
| Bcl3 | -0.278 | 0.129 | 0.0982 | 1 | NM_033601.3:1620 |
| Cfl1 | -0.284 | 0.0556 | 0.00695 | 1 | NM_007687.5:978 |
| Traf6 | -0.284 | 0.225 | 0.276 | 1 | NM_009424.2:980 |
| Ltb4r2 | -0.284 | 0.319 | 0.423 | 1 | NM_020490.2:788 |
| Il6st | -0.285 | 0.191 | 0.211 | 1 | NM_010560.2:2325 |
| Ifna1 | -0.287 | 0.531 | 0.617 | 1 | NM_010502.2:354 |
| Ifit1 | -0.288 | 0.315 | 0.413 | 1 | NM_008331.2:890 |
| Hmgb1 | -0.289 | 0.104 | 0.0499 | 1 | NM_010439.3:1574 |
| Socs3 | -0.289 | 0.196 | 0.215 | 1 | NM_007707.2:585 |
| Itga5 | -0.289 | 0.237 | 0.289 | 1 | NM_010577.3:2446 |
| C4a | -0.289 | 0.272 | 0.348 | 1 | NM_011413.2:56 |
| Cd83 | -0.291 | 0.223 | 0.263 | 1 | NM_009856.2:1624 |
| Vtn | -0.293 | 0.127 | 0.082 | 1 | NM_011707.2:1385 |
| Aire | -0.296 | 0.707 | 0.697 | 1 | NM_009646.1:656 |
| Ctnnb1 | -0.297 | 0.166 | 0.147 | 1 | NM_007614.2:2975 |
| Gpr183 | -0.297 | 0.236 | 0.276 | 1 | NM_183031.2:238 |
| Syk | -0.299 | 0.16 | 0.135 | 1 | NM_011518.2:3970 |
| Klrd1 | -0.3 | 0.17 | 0.153 | 1 | NM_010654.2:434 |
| Il1rap | -0.302 | 0.186 | 0.179 | 1 | NM_008364.2:2415 |
| Cxcl12 | -0.303 | 0.21 | 0.222 | 1 | NM_021704.3:259 |
| Ptprc | -0.305 | 0.16 | 0.13 | 1 | NM_011210.3:2320 |
| Nr3c1 | -0.305 | 0.164 | 0.135 | 1 | NM_008173.3:1800 |
| Csf2rb | -0.306 | 0.197 | 0.195 | 1 | NM_007780.4:4185 |
| Chi3l3 | -0.306 | 0.267 | 0.315 | 1 | NM_009892.1:1196 |
| Cd36 | -0.308 | 0.151 | 0.111 | 1 | NM_007643.3:1520 |
| Cysltr2 | -0.309 | 0.132 | 0.0796 | 1 | NM_001162412.1:1283 |
| Map4k4 | -0.309 | 0.149 | 0.107 | 1 | NM_008696.2:3425 |
| Cd82 | -0.31 | 0.108 | 0.0451 | 1 | NM_001271430.1:186 |
| C7 | -0.31 | 0.293 | 0.35 | 1 | XM_356827.6:215 |
| Csf1 | -0.313 | 0.182 | 0.161 | 1 | NM_001113530.1:833 |
| Cxcr4 | -0.315 | 0.13 | 0.0726 | 1 | NM_009911.3:704 |
| H2-DMa | -0.315 | 0.165 | 0.128 | 1 | NM_010386.3:530 |
| Il17re | -0.315 | 0.167 | 0.132 | 1 | NM_001034029.1:130 |
| Icam2 | -0.316 | 0.233 | 0.248 | 1 | NM_010494.1:375 |
| Lair1 | -0.317 | 0.209 | 0.203 | 1 | NM_001113474.1:1865 |
| Klrc2 | -0.317 | 0.289 | 0.335 | 1 | NM_001098669.1:250 |
| Relb | -0.318 | 0.0975 | 0.031 | 1 | NM_009046.2:2013 |
| H2-Aa | -0.321 | 0.127 | 0.065 | 1 | NM_010378.2:450 |
| Cd40lg | -0.323 | 0.23 | 0.233 | 1 | NM_011616.2:600 |
| Il1b | -0.324 | 0.241 | 0.25 | 1 | NM_008361.3:1120 |
| Nos2 | -0.325 | 0.096 | 0.0275 | 1 | NM_010927.3:3715 |
| Cd48 | -0.325 | 0.156 | 0.106 | 1 | NM_007649.4:30 |
| Ctsg | -0.325 | 0.736 | 0.682 | 1 | NM_007800.1:785 |
| Socs1 | -0.329 | 0.209 | 0.191 | 1 | NM_009896.2:1020 |
| Thy1 | -0.331 | 0.176 | 0.134 | 1 | NM_009382.3:425 |
| Ahr | -0.333 | 0.16 | 0.106 | 1 | NM_013464.4:1327 |
| Iigp1 | -0.338 | 0.334 | 0.369 | 1 | NM_021792.3:2335 |
| Cxcr1 | -0.338 | 0.373 | 0.416 | 1 | NM_178241.4:860 |
| Itga4 | -0.339 | 0.224 | 0.204 | 1 | NM_010576.3:6600 |
| Pou2f2 | -0.341 | 0.0875 | 0.0177 | 1 | NM_001163554.1:2422 |
| C1ra.1 | -0.345 | 0.196 | 0.154 | 1 | NM_023143.3:1923 |
| Mef2c_Mm | -0.346 | 0.229 | 0.205 | 1 | NM_025282.2:1155 |
| Jun | -0.347 | 0.193 | 0.146 | 1 | NM_010591.2:2212 |
| Daxx | -0.348 | 0.161 | 0.0962 | 1 | NM_007829.3:1215 |
| Mafk | -0.349 | 0.153 | 0.0849 | 1 | NM_010757.2:1065 |
| Cd247 | -0.35 | 0.18 | 0.123 | 1 | NM_001113391.2:215 |
| Muc1 | -0.353 | 0.197 | 0.148 | 1 | NM_013605.1:1445 |
| Ccr9 | -0.353 | 0.265 | 0.254 | 1 | NM_009913.6:820 |
| Ccl12 | -0.355 | 0.382 | 0.405 | 1 | NM_011331.2:56 |
| Pecam1 | -0.357 | 0.226 | 0.19 | 1 | NM_008816.2:1100 |
| Lif | -0.358 | 0.131 | 0.0517 | 1 | NM_008501.2:3435 |
| Itgax | -0.359 | 0.175 | 0.11 | 1 | NM_021334.2:3773 |
| Nod2 | -0.361 | 0.259 | 0.236 | 1 | NM_145857.2:2890 |
| Tnfsf11 | -0.361 | 0.432 | 0.45 | 1 | NM_011613.3:615 |
| Bst2 | -0.362 | 0.161 | 0.0877 | 1 | NM_198095.2:468 |
| Msr1 | -0.362 | 0.172 | 0.104 | 1 | NM_001113326.1:555 |
| Zeb1 | -0.362 | 0.196 | 0.138 | 1 | NM_011546.2:4150 |
| Ikzf3 | -0.363 | 0.254 | 0.226 | 1 | NM_011771.1:675 |
| Mme | -0.364 | 0.177 | 0.109 | 1 | NM_008604.3:285 |
| Cd4 | -0.365 | 0.189 | 0.126 | 1 | NM_013488.2:950 |
| Il2rg | -0.369 | 0.176 | 0.103 | 1 | NM_013563.3:1226 |
| Tgfb1 | -0.371 | 0.129 | 0.0454 | 1 | NM_011577.1:1470 |
| Ly86 | -0.372 | 0.173 | 0.0972 | 1 | NM_010745.2:725 |
| Cd74 | -0.374 | 0.0555 | 0.00254 | 1 | NM_001042605.1:391 |
| Lilra5 | -0.374 | 0.172 | 0.0959 | 1 | NM_001081239.2:994 |
| Cxcr5 | -0.375 | 0.327 | 0.316 | 1 | NM_007551.2:1648 |
| Tnf | -0.376 | 0.401 | 0.402 | 1 | NM_013693.1:1135 |
| Tyrobp | -0.378 | 0.154 | 0.0704 | 1 | NM_011662.2:130 |
| Fcer1g | -0.379 | 0.16 | 0.0765 | 1 | NM_010185.4:264 |
| H2-DMb2 | -0.381 | 0.176 | 0.097 | 1 | NM_010388.4:195 |
| Ifngr2 | -0.382 | 0.136 | 0.0483 | 1 | NM_008338.3:797 |
| Arhgdib | -0.383 | 0.121 | 0.0342 | 1 | NM_007486.4:280 |
| Tlr2 | -0.383 | 0.14 | 0.0521 | 1 | NM_011905.2:255 |
| Cfb | -0.387 | 0.201 | 0.127 | 1 | NM_008198.2:1685 |
| Ccl6 | -0.388 | 0.153 | 0.0642 | 1 | NM_009139.2:825 |
| Lcp2 | -0.389 | 0.185 | 0.103 | 1 | NM_010696.3:2605 |
| Cxcl15 | -0.39 | 0.224 | 0.157 | 1 | NM_011339.2:419 |
| Sell | -0.39 | 0.237 | 0.175 | 1 | NM_001164059.1:664 |
| C8b | -0.391 | 0.142 | 0.0512 | 1 | NM_133882.2:1200 |
| Il18rap | -0.391 | 0.17 | 0.0829 | 1 | NM_010553.2:2055 |
| Abcb1a | -0.391 | 0.236 | 0.173 | 1 | NM_011076.1:2600 |
| Tapbp | -0.392 | 0.196 | 0.115 | 1 | NM_009318.2:2195 |
| Ccl21a | -0.392 | 0.2 | 0.122 | 1 | NM_011124.4:170 |
| Cxcl2 | -0.392 | 0.392 | 0.373 | 1 | NM_009140.2:765 |
| Cd80 | -0.394 | 0.135 | 0.0434 | 1 | NM_009855.2:210 |
| Cd274 | -0.395 | 0.209 | 0.133 | 1 | NM_021893.2:515 |
| Cd22 | -0.395 | 0.241 | 0.176 | 1 | NM_001043317.2:865 |
| H2-Ab1 | -0.399 | 0.109 | 0.0213 | 1 | NM_207105.2:164 |
| Cd3d | -0.399 | 0.175 | 0.085 | 1 | NM_013487.2:289 |
| Aicda | -0.399 | 0.436 | 0.412 | 1 | NM_009645.2:552 |
| Emr1 | -0.402 | 0.144 | 0.0496 | 1 | NM_010130.1:995 |
| Tfrc | -0.406 | 0.184 | 0.0918 | 1 | NM_011638.3:1930 |
| Ifi44 | -0.409 | 0.272 | 0.207 | 1 | NM_133871.2:990 |
| Tnfrsf13b | -0.413 | 0.215 | 0.127 | 1 | NM_021349.1:340 |
| Tnfrsf1b | -0.415 | 0.193 | 0.0988 | 1 | NM_011610.3:3270 |
| Tal1 | -0.415 | 0.231 | 0.147 | 1 | NM_011527.2:2490 |
| Fos | -0.415 | 0.238 | 0.156 | 1 | NM_010234.2:1330 |
| Cd79b | -0.418 | 0.278 | 0.207 | 1 | NM_008339.2:330 |
| Mapkapk5 | -0.421 | 0.0957 | 0.0117 | 1 | XM_990515.1:53 |
| Cd2 | -0.428 | 0.109 | 0.0172 | 1 | NM_013486.2:278 |
| Tnfsf14 | -0.429 | 0.114 | 0.0197 | 1 | NM_019418.2:1060 |
| C2 | -0.429 | 0.216 | 0.118 | 1 | NM_013484.2:2359 |
| Kir3dl1 | -0.433 | 0.78 | 0.608 | 1 | NM_177749.3:942 |
| Fcgr1 | -0.437 | 0.149 | 0.0426 | 1 | NM_010186.5:185 |
| Ptger1 | -0.438 | 0.225 | 0.123 | 1 | NM_013641.2:928 |
| Ager | -0.44 | 0.153 | 0.0449 | 1 | NM_007425.2:361 |
| Cdh5 | -0.44 | 0.271 | 0.18 | 1 | NM_009868.3:1615 |
| Plaur | -0.442 | 0.16 | 0.0509 | 1 | NM_011113.3:1085 |
| Ccl25 | -0.442 | 0.225 | 0.121 | 1 | NM_009138.3:626 |
| Ccr7 | -0.444 | 0.166 | 0.0556 | 1 | NM_007719.2:755 |
| Ifit3 | -0.444 | 0.349 | 0.273 | 1 | NM_010501.1:1290 |
| Il9 | -0.445 | 0.139 | 0.0332 | 1 | NM_008373.1:39 |
| Cd5 | -0.446 | 0.232 | 0.127 | 1 | NM_007650.3:1395 |
| Il17a | -0.447 | 0.369 | 0.292 | 1 | NM_010552.3:205 |
| Lck | -0.449 | 0.201 | 0.0894 | 1 | NM_010693.2:1180 |
| Cd9 | -0.451 | 0.146 | 0.0368 | 1 | NM_007657.3:620 |
| Cd69 | -0.453 | 0.221 | 0.109 | 1 | NM_001033122.3:91 |
| Il33 | -0.454 | 0.21 | 0.0966 | 1 | NM_133775.1:1011 |
| Klra8 | -0.467 | 0.216 | 0.097 | 1 | NM_010650.3:842 |
| App | -0.471 | 0.22 | 0.0991 | 1 | NM_007471.2:511 |
| Cd3e | -0.476 | 0.214 | 0.0904 | 1 | NM_007648.4:380 |
| Il13 | -0.478 | 0.452 | 0.35 | 1 | NM_008355.2:425 |
| Itgb1 | -0.48 | 0.21 | 0.0846 | 1 | NM_010578.1:1855 |
| Il19 | -0.48 | 0.614 | 0.478 | 1 | NM_001009940.1:464 |
| Cx3cr1 | -0.482 | 0.167 | 0.045 | 1 | NM_009987.3:2696 |
| S100a8 | -0.483 | 0.422 | 0.316 | 1 | NM_013650.2:227 |
| Spn | -0.486 | 0.181 | 0.0546 | 1 | NM_001037810.1:726 |
| Itga6 | -0.487 | 0.288 | 0.166 | 1 | NM_008397.3:910 |
| Klra4 | -0.487 | 0.352 | 0.238 | 1 | NM_010649.3:169 |
| Cxcr6 | -0.493 | 0.138 | 0.0233 | 1 | NM_030712.4:650 |
| Clec5a | -0.494 | 0.202 | 0.0703 | 1 | NM_001038604.1:605 |
| Nlrp3 | -0.497 | 0.144 | 0.0258 | 1 | NM_145827.3:2745 |
| Sh2d1a | -0.497 | 0.162 | 0.037 | 1 | NM_011364.3:250 |
| Tnfsf10 | -0.498 | 0.276 | 0.146 | 1 | NM_009425.2:2055 |
| Ctsc | -0.503 | 0.205 | 0.07 | 1 | NM_009982.2:2225 |
| Trem1 | -0.506 | 0.262 | 0.125 | 1 | NM_021406.3:290 |
| Batf | -0.507 | 0.219 | 0.0818 | 1 | NM_016767.2:750 |
| Il12rb2 | -0.512 | 0.141 | 0.022 | 1 | NM_008354.3:1395 |
| Plau | -0.516 | 0.183 | 0.0478 | 1 | NM_008873.2:1950 |
| Ctss | -0.518 | 0.161 | 0.0325 | 1 | NM_021281.2:740 |
| S100a9 | -0.521 | 0.475 | 0.334 | 1 | NM_009114.2:112 |
| Tgfb2 | -0.528 | 0.193 | 0.0517 | 1 | NM_009367.1:1685 |
| Il1rn.1 | -0.53 | 0.195 | 0.0535 | 1 | NM_031167.5:224 |
| Ltb | -0.534 | 0.189 | 0.0475 | 1 | NM_008518.2:163 |
| Ptgs2 | -0.534 | 0.234 | 0.0847 | 1 | NM_011198.3:675 |
| Itgb2 | -0.536 | 0.195 | 0.0512 | 1 | NM_008404.4:2542 |
| Bst1 | -0.539 | 0.157 | 0.0266 | 1 | NM_009763.3:542 |
| Klrc1 | -0.541 | 0.111 | 0.00811 | 1 | NM_001136068.1:68 |
| Cd44 | -0.546 | 0.173 | 0.0346 | 1 | NM_009851.2:3075 |
| Tnfrsf13c | -0.551 | 0.314 | 0.154 | 1 | NM_028075.2:1170 |
| Nt5e | -0.555 | 0.327 | 0.165 | 1 | NM_011851.3:1600 |
| Itgam | -0.557 | 0.27 | 0.108 | 1 | NM_001082960.1:3025 |
| Il23a | -0.57 | 0.439 | 0.264 | 1 | NM_031252.1:360 |
| Clec4e | -0.603 | 0.291 | 0.107 | 1 | NM_019948.2:685 |
| Cybb | -0.613 | 0.181 | 0.0275 | 1 | NM_007807.2:1535 |
| Ccl5 | -0.614 | 0.171 | 0.0232 | 1 | NM_013653.1:165 |
| Itgal | -0.614 | 0.211 | 0.0437 | 1 | NM_008400.2:950 |
| Hcst | -0.616 | 0.25 | 0.0692 | 1 | NM_011827.3:166 |
| Il1a | -0.618 | 0.217 | 0.0463 | 1 | NM_010554.4:225 |
| Ccrl1 | -0.625 | 0.344 | 0.144 | 1 | NM_145700.2:780 |
| Btnl1 | -0.626 | 0.477 | 0.259 | 1 | NM_001111094.1:1740 |
| Klrb1 | -0.627 | 0.536 | 0.307 | 1 | NM_001099918.1:327 |
| Gzmb | -0.631 | 0.158 | 0.0161 | 1 | NM_013542.2:1020 |
| Areg | -0.641 | 0.419 | 0.201 | 1 | NM_009704.3:698 |
| Cx3cl1 | -0.642 | 0.23 | 0.0495 | 1 | NM_009142.3:125 |
| Ms4a1 | -0.642 | 0.353 | 0.143 | 1 | NM_007641.5:166 |
| Prf1 | -0.645 | 0.204 | 0.0343 | 1 | NM_011073.2:1350 |
| Gata3 | -0.648 | 0.261 | 0.068 | 1 | NM_008091.3:1943 |
| Icam1 | -0.661 | 0.195 | 0.0274 | 1 | NM_010493.2:2195 |
| Il25 | -0.676 | 0.425 | 0.187 | 1 | NM_080729.2:649 |
| Slamf1 | -0.678 | 0.488 | 0.237 | 1 | NM_013730.4:95 |
| Klrk1 | -0.681 | 0.188 | 0.0221 | 1 | NM_001083322.1:144 |
| Vcam1 | -0.687 | 0.254 | 0.0535 | 1 | NM_011693.2:1440 |
| Ccl3 | -0.699 | 0.174 | 0.016 | 1 | NM_011337.1:60 |
| Ccr3 | -0.702 | 0.225 | 0.0353 | 1 | NM_009914.4:2290 |
| Cd7 | -0.722 | 0.269 | 0.0553 | 1 | NM_009854.1:234 |
| Ifng | -0.727 | 0.397 | 0.141 | 1 | NM_008337.1:95 |
| Il1rn | -0.737 | 0.292 | 0.0653 | 1 | NM_031167.4:1895 |
| Il5 | -0.739 | 0.271 | 0.0525 | 1 | NM_010558.1:177 |
| Il2rb | -0.742 | 0.087 | 0.00104 | 1 | NM_008368.3:2365 |
| Cd28 | -0.774 | 0.277 | 0.0494 | 1 | NM_007642.4:3304 |
| Gp1bb | -0.781 | 0.416 | 0.134 | 1 | NM_010327.2:1555 |
| Tnfrsf9 | -0.795 | 0.415 | 0.128 | 1 | NM_001077508.1:1590 |
| Rag1 | -0.807 | 0.796 | 0.368 | 1 | NM_009019.2:1945 |
| Alox12 | -0.809 | 0.307 | 0.0579 | 1 | NM_007440.4:1426 |
| Cd160 | -0.831 | 0.129 | 0.00299 | 1 | NM_001163496.1:1403 |
| Gzma | -0.836 | 0.158 | 0.00608 | 1 | NM_010370.2:188 |
| Ccr4 | -0.839 | 0.297 | 0.0476 | 1 | NM_009916.2:1670 |
| C8g | -0.843 | 0.326 | 0.061 | 1 | NM_027062.1:770 |
| Klra7 | -0.866 | 0.188 | 0.0101 | 1 | NM_001110323.1:250 |
| Ccl20 | -0.896 | 0.631 | 0.228 | 1 | NM_016960.1:120 |
| Eomes | -0.903 | 0.333 | 0.0535 | 1 | NM_010136.2:2665 |
| Cxcl10 | -0.924 | 0.21 | 0.0117 | 1 | NM_021274.1:115 |
| Xcl1 | -0.944 | 0.0836 | 0.00035 | 1 | NM_008510.1:103 |
| Cxcl3 | -0.963 | 0.323 | 0.0407 | 1 | NM_203320.2:275 |
| Tbx21 | -0.968 | 0.172 | 0.00495 | 1 | NM_019507.1:625 |
| Cxcl1 | -0.989 | 0.257 | 0.0183 | 1 | NM_008176.1:560 |
| Hmgb2 | -1.02 | 0.407 | 0.0663 | 1 | NM_008252.3:1667 |
| Il11 | -1.04 | 0.444 | 0.0787 | 1 | NM_008350.2:285 |
| Il17b | -1.04 | 0.525 | 0.119 | 1 | NM_019508.1:346 |
| Cd226 | -1.06 | 0.35 | 0.0391 | 1 | NM_001039149.1:733 |
| Nox1 | -1.09 | 0.3 | 0.0223 | 1 | NM_172203.1:475 |
| Il4 | -1.09 | 0.37 | 0.0421 | 1 | NM_021283.1:345 |
| Ccl4 | -1.1 | 0.279 | 0.017 | 1 | NM_013652.1:140 |
| Il17f | -1.17 | 0.326 | 0.0228 | 1 | NM_145856.2:625 |
| C8a | -1.22 | 0.435 | 0.0484 | 1 | NM_146148.1:1350 |
| Sele | -1.23 | 0.256 | 0.00859 | 1 | NM_011345.2:2575 |
| Itga2b | -1.26 | 0.423 | 0.0409 | 1 | NM_010575.2:461 |
| Ppbp | -1.29 | 0.517 | 0.0667 | 1 | NM_023785.2:225 |
| Pla2g2a | -1.35 | 0.625 | 0.0966 | 1 | NM_001082531.1:220 |
| Ccl26 | -1.47 | 1.05 | 0.236 | 1 | NM_001013412.2:140 |
| Defa-rs1 | -1.74 | 0.916 | 0.131 | 1 | NM_007844.2:338 |
| Camp | -3.05 | 0.804 | 0.0192 | 1 | NM_009921.2:355 |
