# Supplementary figures and images for: Orientia tsutsugamushi selectively stimulates the C-type lectin receptor Mincle and type 1-skewed proinflammatory immune responses
Source: PLoS Pathog. 2021 Jul 28;17(7):e1009782. doi: 10.1371/journal.ppat.1009782 (PMC8351992; doi:10.1371/journal.ppat.1009782)

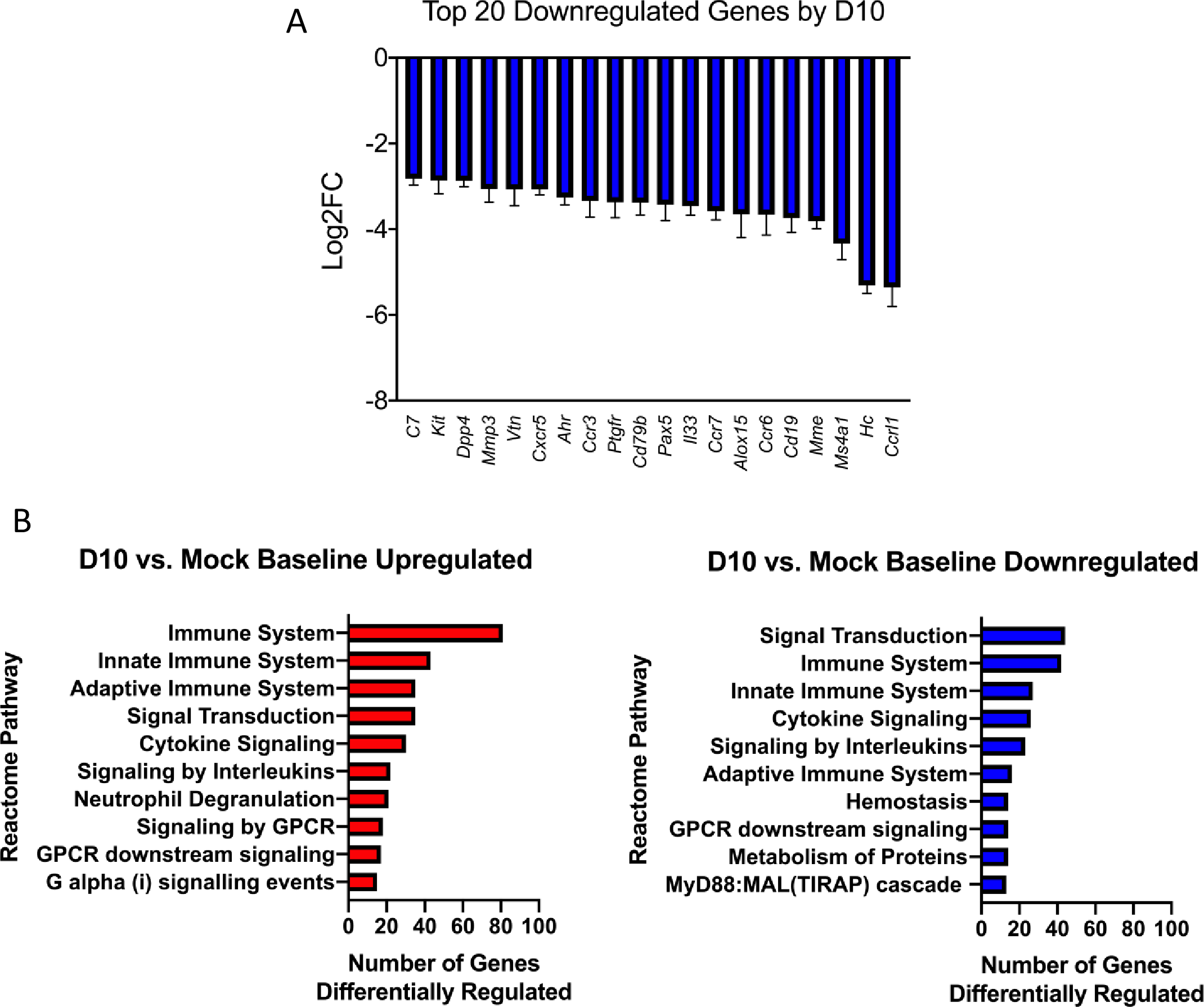

Supplement: S1 Fig — Nanostring gene profiling analysis was performed on RNA isolated from lung tissue homogenates of lethally infected mice at D10 and compared with the mock controls. (A) Shown are the bottom 20 most downregulated genes by Log2FC. (B) Significantly upregulated or downregulated genes were input to the String database and analyzed by the Reactome pathway. Graphs are shown as mean ± SEM. Differential expression analysis was performed utilizing the Benjamini-Yekutieli test for significance. (TIF) [file ppat.1009782.s005.tif]

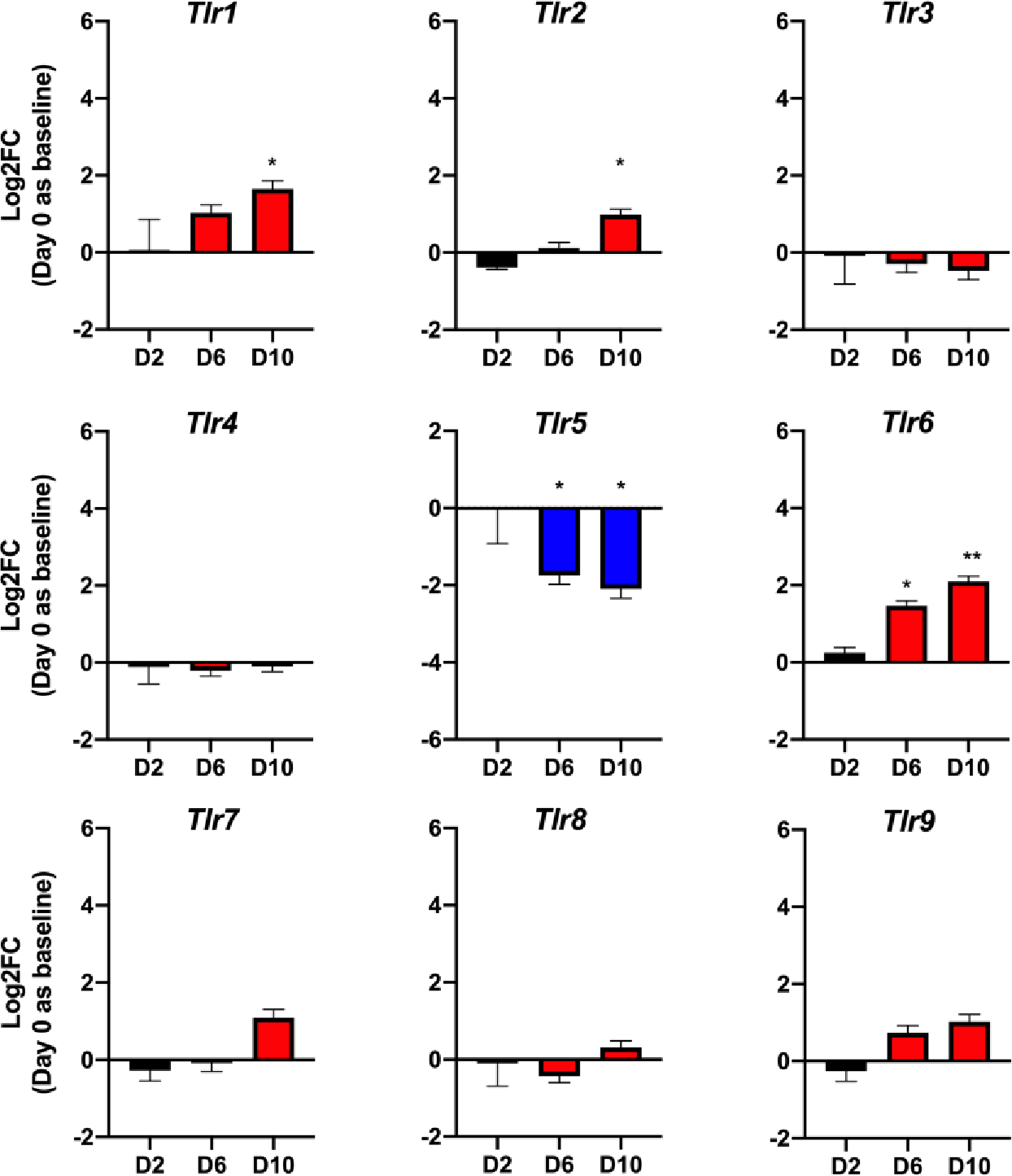

Supplement: S2 Fig — Nanostring gene profiling analysis was performed on RNA isolated from lung tissue homogenates of lethally infected mice at D2, D6, and D10, respectively, and compared with the mock controls. We then parsed our data to examine expression of Tlr genes. Scale is Log2Fold change compared to mock. Graphs are shown as mean ± SEM. Significance was determined utilizing the Benjamini-Yekutieli test. *, p < 0.05, ** p < 0.01. (TIF) [file ppat.1009782.s006.tif]

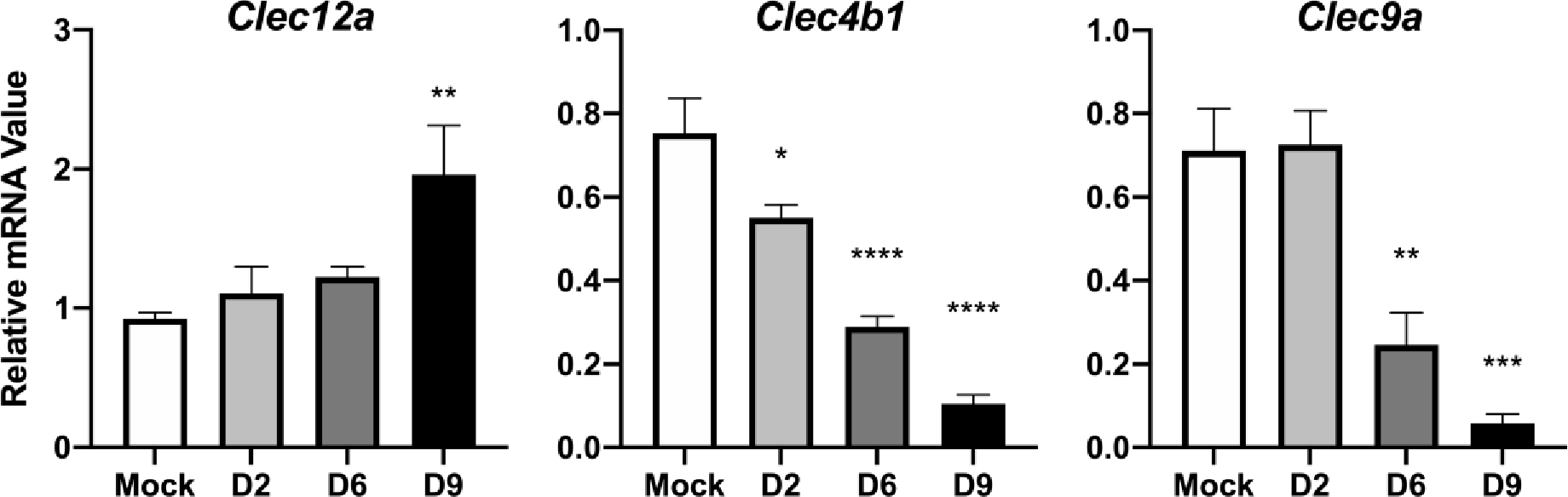

Supplement: S3 Fig — Whole lung tissue homogenates from lethally infected mice were measured for expression of indicated CLRs via qRT-PCR. All data are presented relative to GAPDH values and shown as mean ± SEM. Three independent mouse infection experiments were performed with similar trends; representative data are shown. One-way ANOVA with Dunnett’s multiple comparison test was used for statistical analysis, with mock samples used as a reference. *, p < 0.05, ** p < 0.01, *** p < 0.001, **** p < 0.0001. (TIF) [file ppat.1009782.s007.tif]

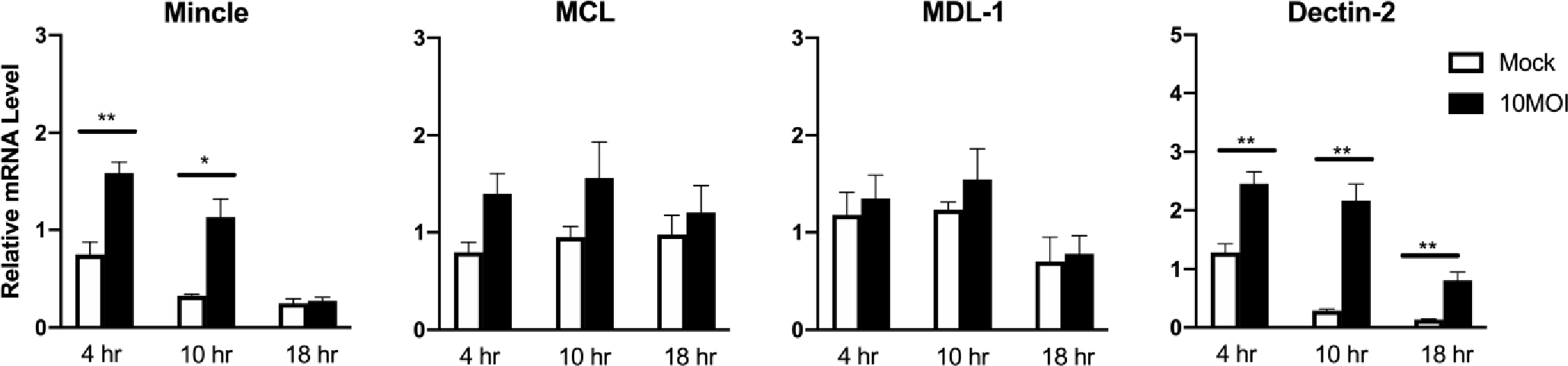

Supplement: S4 Fig — Bone marrow-derived neutrophils of C57BL/6J mice were exposed to live bacteria (10 MOI). mRNA levels of select CLRs were analyzed via qRT-PCR. Data are presented relative to GAPDH values and shown as mean ± SEM. Unpaired t-test was used for statistical analysis, with Mock samples used as a reference. *, p < 0.05, ** p < 0.01. (TIF) [file ppat.1009782.s008.tif]

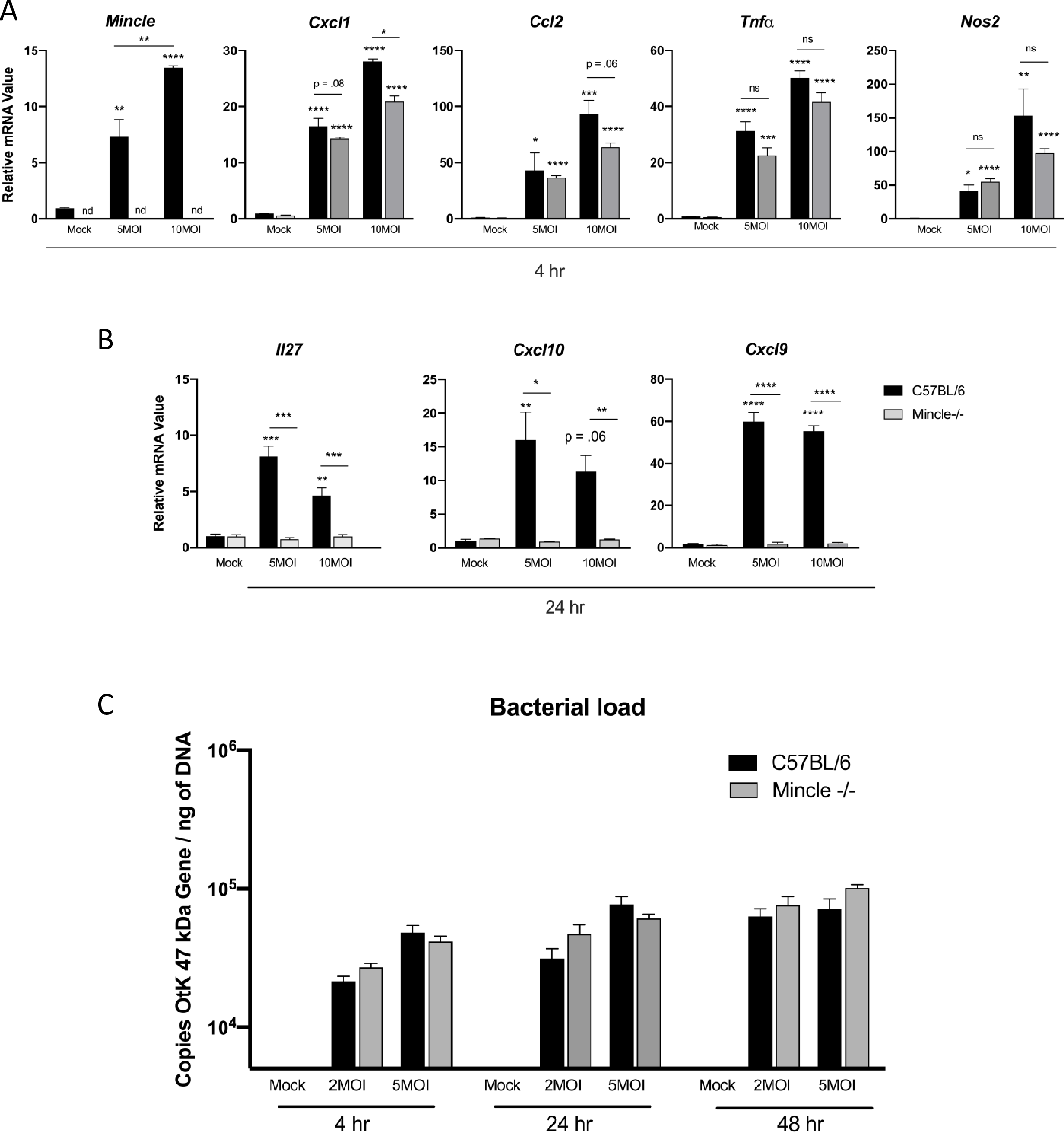

Supplement: S5 Fig — Bone marrow-derived WT or Mincle-/- MΦ were exposed to live bacteria (MOI 5, or 10). qRT-PCR analyses of indicated genes at (A) 4 hr and (B) 24 hr post-infection are presented with data presented relative to GAPDH and shown as mean ± SEM. One-way ANOVA with Dunnett’s multiple comparison test was performed for treatment groups within the WT or Mincle-/- MΦ background, respectively. Unpaired t-test was utilized for comparison between infected WT and Mincle-/- MΦs. (C) Bacterial growth in infected MΦs (MOI 2 or 5) was analyzed at 4, 24, and 48 hr. Bacterial loads were determined by qPCR. Data are presented as the copy number of O. tsutsugamushi 47-kDa gene copy per ng of DNA. Unpaired t-test was used for statistical analysis. *, p < 0.05; **, p < 0.01; ***, p < 0.001; ****, p < 0.0001. (TIF) [file ppat.1009782.s009.tif]

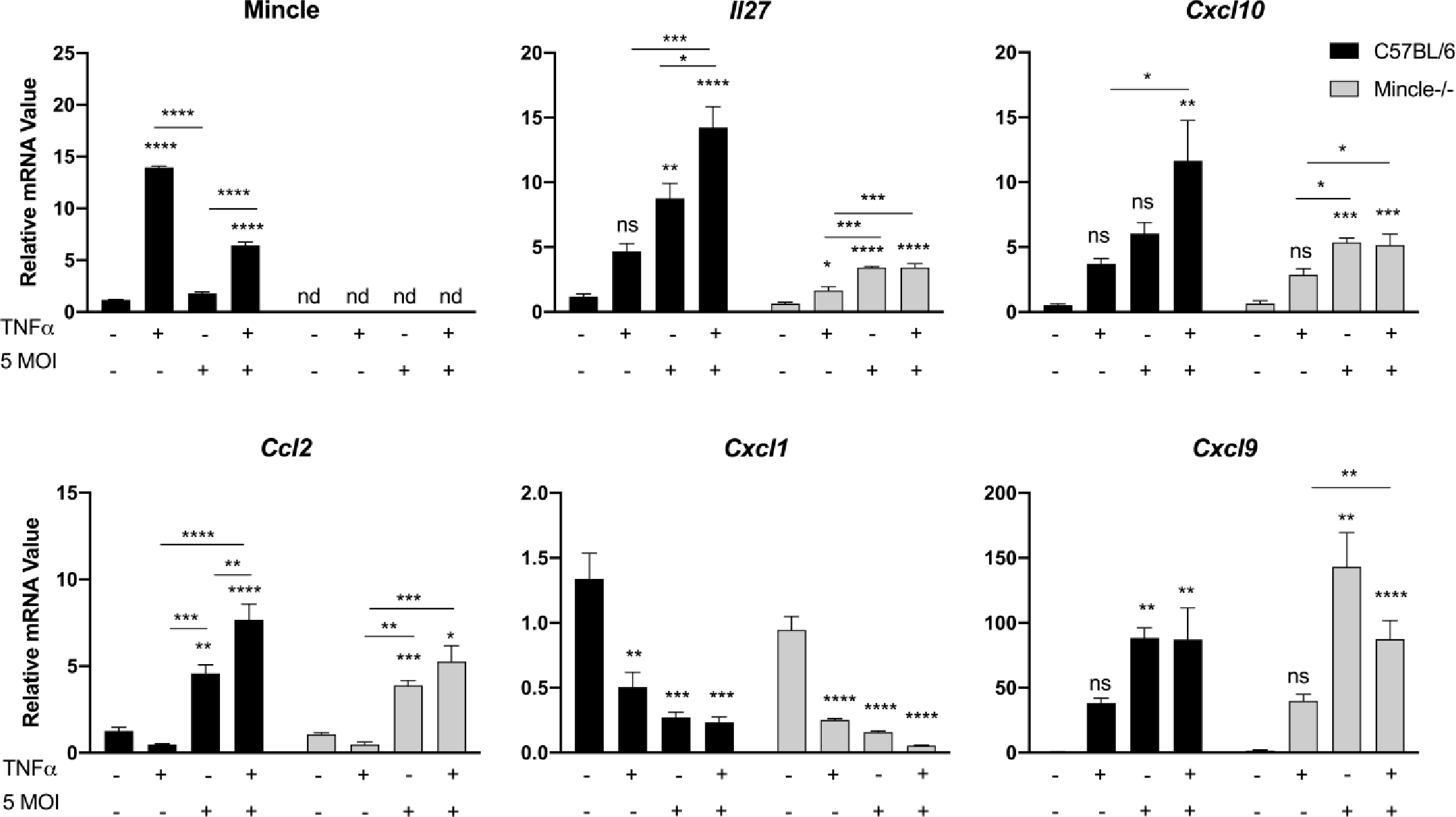

Supplement: S6 Fig — Bone marrow-derived WT or Mincle-/- MΦ were primed with TNFα (25 ng/ml for 30 min) prior to infection with live bacteria (5 MOI). qRT-PCR analyses of indicated genes (relative to GAPDH) at 24 hr post-infection are presented; data are shown as mean ± SEM. One-way ANOVA with Dunnett’s multiple comparison test was performed for treatment groups within the WT or Mincle-/- MΦ backgrounds, respectively. Unpaired t-test was utilized for comparison between infected WT and Mincle-/- MΦs. *, p < 0.05; **, p < 0.01; ***, p < 0.001; ****, p < 0.0001. (TIF) [file ppat.1009782.s010.tif]
